# CRISPR/Cas9 therapeutics: progress and prospects

**DOI:** 10.1038/s41392-023-01309-7

**Published:** 2023-01-16

**Authors:** Tianxiang Li, Yanyan Yang, Hongzhao Qi, Weigang Cui, Lin Zhang, Xiuxiu Fu, Xiangqin He, Meixin Liu, Pei-feng Li, Tao Yu

**Affiliations:** 1 Institute for Translational Medicine, The Affiliated Hospital of Qingdao University, No. 38 Dengzhou Road, 266021 Qingdao, People’s Republic of China; 2 Department of Immunology, School of Basic Medicine, Qingdao University, 266021 Qingdao, People’s Republic of China; 3 Department of Cardiology, People’s Hospital of Rizhao, No. 126 Taian Road, 276827 Rizhao, People’s Republic of China; 4 Department of Microbiology, Linyi Center for Disease Control and Prevention, 276000 Linyi, People’s Republic of China; 5 Department of Cardiac Ultrasound, The Affiliated Hospital of Qingdao University, 266000 Qingdao, People’s Republic of China

**Keywords:** Molecular medicine, Gene delivery

## Abstract

Clustered regularly interspaced short palindromic repeats (CRISPR)/CRISPR-associated protein 9 (Cas9) gene-editing technology is the ideal tool of the future for treating diseases by permanently correcting deleterious base mutations or disrupting disease-causing genes with great precision and efficiency. A variety of efficient Cas9 variants and derivatives have been developed to cope with the complex genomic changes that occur during diseases. However, strategies to effectively deliver the CRISPR system to diseased cells in vivo are currently lacking, and nonviral vectors with target recognition functions may be the focus of future research. Pathological and physiological changes resulting from disease onset are expected to serve as identifying factors for targeted delivery or targets for gene editing. Diseases are both varied and complex, and the choice of appropriate gene-editing methods and delivery vectors for different diseases is important. Meanwhile, there are still many potential challenges identified when targeting delivery of CRISPR/Cas9 technology for disease treatment. This paper reviews the current developments in three aspects, namely, gene-editing type, delivery vector, and disease characteristics. Additionally, this paper summarizes successful examples of clinical trials and finally describes possible problems associated with current CRISPR applications.

## Introduction

Gene editing is a technology that precisely modifies the genome sequence to induce insertions, deletions, or base substitutions in the genome.^1,2^ Many diseases are accompanied by changes in gene expression in vivo, particularly some genetic diseases caused by mutations in a single gene, and gene-editing technology is expected to control the occurrence of diseases at the genetic level.^3^ To date, gene-editing technology has undergone three main generations of development: the first generation of gene-editing technology was zinc-finger nucleases (ZFNs); the second generation was transcription activator-like effector nucleases (TALENs); and the most widely used third generation gene-editing technology is clustered regularly interspaced short palindromic repeats (CRISPR)/CRISPR-associated protein 9 (Cas).^4^ Unlike ZFNs and TALENs, which use proteins to target DNA strands, CRISPR technology directs Cas proteins to a specified location in the genome by changing the base sequence of a small segment of guide RNA, thus improving the efficiency of gene editing and expanding the applicability of gene-editing technology.^5^

CRISPR/ Cas9 is a highly effective gene-editing tool that is widely used in the scientific community.^6^ The CRISPR/Cas9 system evolved naturally in bacteria and archaea as a defense mechanism against phage infection and plasmid transfer.^7,8^ Bacteria or archaea acquire a segment of their DNA sequence to insert into the CRISPR spacer region when first infiltrated by an exogenous phage or plasmid. If reinfected with homologous DNA, the bacterium will initiate transcription of the CRISPR region. After a series of processing and maturation processes to generate a single guide RNA (sgRNA), the sgRNA guides Cas9 to shear the DNA strand that disrupts the homologous spacer region. The recognition process of the sgRNA requires the involvement of protospacer-adjacent motifs (PAMs), a short guanine-enriched sequence.^9^ The preferred PAM by *Streptococcus pyogenes* Cas9 (SpCas9) is NGG, which is common in the genomes of most organisms, thereby facilitating the use of CRISPR technology across the fields of plant and animal science, together with biomedicine.^10–14^ By changing the nucleotide sequence of a small segment of guide RNA, CRISPR/Cas9 allows the accurate targeting of almost any desired genomic locus for the purpose of correcting disease-causing mutations or silencing genes associated with disease onset.^5,15^ However, some highly chromatinized regions in the genome may not be accessible to CRISPR/Cas9. Promising applications for this technology include the treatment of cancers, cardiovascular diseases, sickle cell anemia, and neurodegenerative disease.^16–19^

Wild-type Cas9 only cuts double-stranded DNA to form double-strand breaks (DSBs), which are repaired through DNA repair mechanisms, namely, homology-directed repair (HDR) and nonhomologous end joining (NHEJ).^20–22^ The base sequence of the original gene is damaged, resulting in inactivation, but the inactivation of a single deleterious gene cannot address the complex processes of all disease events.^23^ Therefore, researchers searched for possible ways to modify Cas9 by elucidating the physicochemical structure of Cas9, the mechanism of action by which Cas9 cleaves double chains, and other properties. They endowed Cas9 with new functions by mutating the structural domain of Cas9 and introducing effectors, including transcriptional regulatory tools such as dead Cas9 (dCas9) effectors and single-base substitution tools such as cytosine base editors (CBEs), adenine base editors (ABEs), and prime editors (PEs). Moreover, RNA recognition and cleavage functions can be performed by Cas13a isolated from *Leptotrichia shahii.*^24–28^ These Cas9 variants and derivatives enrich the gene-editing paradigm and can be adapted to additional types of diseases.

Although several experiments have documented the use of gene-editing technology to modify cells in vitro for return to the body to treat some diseases, this approach is not applicable to most disease types. Achieving stable, efficient, and safe delivery in vivo is a challenge that must be overcome before CRISPR technology becomes a common treatment modality. CRISPR systems such as plasmid DNA (pDNA), mRNA and ribonucleoproteins (RNPs) are subject to degradation and immune clearance in vivo after direct delivery and therefore require the help of delivery vectors.^29^ Adeno-associated virus (AAV) vectors are not suitable for application in most diseases because of the drawbacks of a limited loading capacity, a lack of specific targeting ability and inability to integrate into the host genome.^30^ Nonviral vectors have been a hot topic of research in recent years, where lipid nanoparticles have been used in the clinic for the delivery of CRISPR gene drugs.^31^ Polymeric nanoparticles, biomimetic nanomaterials, and exosomes have also shown potential for the delivery of CRISPR systems in animal experiments.^32^ Further research and development are needed to apply nonviral vectors to a wide range of clinical applications.

Each disease has different characteristics, and our aim is not to develop a universal delivery vehicle but to develop multiple vehicles applicable to different types of diseases. Therefore, studying the pathogenesis of diseases and the pathological characteristics of disease cells and tissues and constructing environment-responsive and ligand-recognizing nanoparticles based on these characteristics will further enrich gene-targeting drugs in diseased tissues.^33^ In addition, exosomes and cell membranes from immune cells or diseased organs can effectively avoid immune clearance, and the abundant membrane proteins on the surface enable gene-targeting drugs to be delivered to diseased cells.

In this review, we discuss the development of CRISPR technology and summarize the various types of gene-editing tools that have been developed in recent years. Delivery systems for CRISPR systems in the body are also summarized, with a focus on developing new systems more suitable for different diseases, and finally, the review addresses a collection of problems that may arise when applying CRISPR technology to treat diseases and the corresponding strategies. In conclusion, this approach has positive implications for providing the most effective gene therapy modalities for different diseases.

## Discovery and development of CRISPR technology

**Fig. 1:**
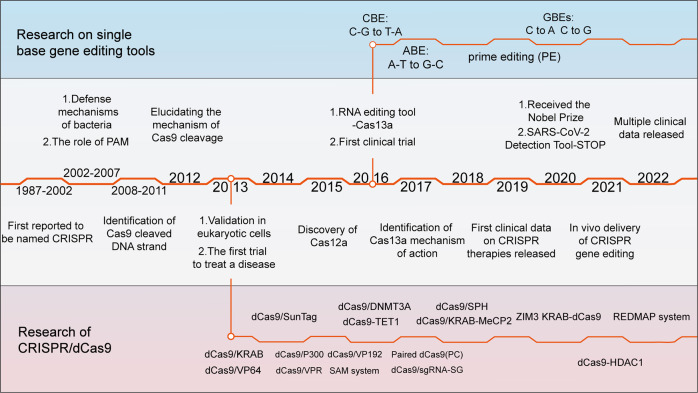
Timeline of major events in the development of CRISPR/Cas technology and representative Cas9 variants. In 1987, the CRISPR sequence was first reported. The mechanism by which Cas9 cuts DNA double strands was reported in 2012, and Cas9 was subsequently used for gene editing in mammalian cells. Since then, CRISPR technology has developed rapidly, and multiple Cas9 variants with specific functions have been identified. The representative variants are single-base substitution tools (e.g., CBE and PE) and transcriptional regulatory tools (e.g., dCas9-effector). Since 2016, CRISPR-based gene-editing technologies have been successively used in clinical treatment with great success. CRISPR clustered regularly interspaced short palindromic repeats, Cas CRISPR-associated, dCas9 dead Cas9, PAM protospacer-adjacent motifs, CBE cytosine base editors, ABE adenine base editors, GBE glycosylase base editors. (Figure was created with Adobe Illustrator)

CRISPR-related gene-editing technology is currently one of the hottest biological tools. Since 2013, explosive growth has been recorded in the study of CRISPR technology, with tens of thousands of CRISPR-related articles published. In October 2020, the Nobel Prize in Chemistry was awarded to French microbiologist Emmanuelle Charpentier and American biologist Jennifer Doudna for “developing a new approach to genome editing”. The method had been studied by scientists for nearly three decades before it received widespread attention (Fig. 1).

### Early detection using CRISPR technology

#### A special sequence of repeated intervals

Like many great discoveries, the discovery of CRISPR technology was born out of an unexpected event. An unusual sequence identified in the 3′ end structural domain of the *iap* gene was first reported by Nakata et al. in 1987 while studying the *iap* gene of *E. coli*. The sequence consisted of five highly homologous sequences containing 29 nucleotides separated by 32 nucleotides.^34^

Over the next decade, this particular repeat sequence was detected in a variety of bacteria and archaea.^35–39^ In 2002, Janson et al. provided a generalized summary of the specific repeats that have been identified, naming these repeats as a family and using the acronym CRISPR for clustered regularly interspaced short palindromic repeats.^40^ In addition, multiple CRISPR-associated proteins (Cas)-Cas1 to Cas4- have been revealed in previous studies.

#### Bacterial and archaeal defense weapons

In 2005, researchers discovered that the spacer sequences in CRISPR are not unique to each organism.^8^ Mojica et al. found that most of the spacer sequences were derived from exogenous DNA, with only a small fraction unrelated to the outside world, and they found that viruses were more likely to infect cells without homologous spacer sequences.^8^ They conjectured that CRISPR is involved in bacterial resistance to infection by external phages and in plasmid transfer.^11,41^ The conjecture was confirmed 2 years later.^42–44^

When first confronted with phage or plasmid infestation, bacteria containing CRISPR sequences acquire a segment of their DNA sequence, which serves as a spacer region between special repeat sequences. CRISPR RNA (crRNA) then undergoes a series of transcription and maturation processes to produce a single crRNA containing a protospacer sequence of 20 bases that binds to the invading DNA via complementary base pairing.^45,46^ Recognition of the exogenous sequence by crRNA alone does not protect it from the phage; it also must be inactivated by disrupting the exogenous sequence through the cleavage activity of the Cas protein.^47,48^

The CRISPR/Cas family of proteins is divided into two categories based on genomic and protein structure information, and the best-known protein Cas9 is among the Class II CRISPR/Cas systems.^49,50^ Class I is characterized by a large Cas9 protein complex that shears the DNA strand, while Class II requires only a single shearing protein. Cas9 is characterized by the presence of two ribonuclease structural domains, a RuvC-like nuclease domain near the amino terminus and the HNH nuclease domain in the middle of the protein, both of which have the function of cleaving the DNA strand.^51^ Notably, protospacer sequences are not randomly acquired from exogenous sequences but are always accompanied by a guanine-enriched sequence called protospacer-adjacent motifs (PAMs).^43^ Subsequent studies have shown that PAM sequences play an important role in the acquisition of the spacer region, where Cas proteins perform cleavage.^5,15,52^

### Contributions of Charpentier and Doudna

The functional mechanism of CRISPR/Cas9 has been gradually revealed, and natural CRISPR/Cas9 has been rapidly applied to bacterial transformation.^44,53^ In 2011, Siksnys et al. transferred the first CRISPR gene sequence from *Streptococcus thermophilus* to *E. coli*, and the *E. coli* that received the CRISPR gene sequence successfully resisted plasmid transformation, which was the first report that CRISPR/Cas9 functioned in a nonhost bacterium.^54^ This finding suggested that CRISPR/Cas systems can be used as a defense mechanism against external infection and that their hosts are not necessary for the CRISPR system to function.

In 2012, Charpentier and Doudna purified Cas9 from *S. thermophilus* and *Streptococcus pyogenes*, enabling the cleavage of prokaryotic DNA in vitro.^47,55^ They also elucidated the mechanism by which CRISPR/Cas9 works, noting that the cleavage site of Cas9 is controlled by a seed sequence in the crRNA and requires the involvement of PAM. Additionally, by altering the nucleotide sequence of a seed sequence, the system can function as a gene silencer in a variety of situations, providing gene targeting and gene editing by changing a nucleotide seed sequence.

### The boom in CRISPR technology

#### Gene editing in mammalian cells

Previous research on CRISPR/Cas9 has focused on prokaryotic cells, and CRISPR technology started to be used in medicine, agriculture, and other fields in a paper published by Zhang Feng et al. in 2013.^56^ They used human-derived 293 T cells, into which they integrated trans-activating crRNA (tracrRNA), pre-crRNA, host factor ribonuclease (RNase) III, and Cas9 from *S. pyogenes* and added the respective promoters and two nuclear localization signals (NLSs) to ensure the entry of the structure into the nucleus.^47,48,54,57^ This experiment targeted 30 base pairs located before the PAM at the human empty spiracle homeobox 1 (EMX1) locus, and the results showed that cleavage of EMX1 was achieved with the inclusion of at least spCas9, tracrRNA and pre-crRNA. Additionally, the function of Cas9 from *S. thermophilus* was verified by Zhang Feng et al. and produced consistent results.

In another paper published the same year, Church et al. constructed crRNA-tracrRNA fusion transcripts that became single guide RNAs (sgRNAs) and shrank crRNAs to 20 bp.^58^ These studies had significant implications, both confirming that CRISPR motifs function in mammalian cells and simplifying the CRISPR gene-editing system, thereby providing more possibilities for the use of CRISPR.

#### The transcriptional regulatory tool dCas9

The DNA strand cleavage function of Cas9 was elucidated by designing a simple sgRNA segment to guide Cas9 to the target site, but many additional studies on genes have been performed to address functions other than DNA strand cleavage. Qi et al. mutated the RuvC1 and HNH nuclease domains (D10A and H841A) of the wild-type Cas9 mentioned above, causing Cas9 to lose its cleavage enzyme activity.^24^ dCas9 showed efficient gene silencing when sgRNAs were designed for nontemplate DNA strands, while sgRNAs designed for template strands did not effectively silence gene expression. The relative positions of sgRNAs and target gene promoter sequences also had a significant effect on silencing efficiency. Importantly, for the sgRNA targeting promoter sequences, gene silencing occurs regardless of whether the target is the template or nontemplate strand. In July 2013, another study by Qi et al. revealed that dCas9 interacts with effectors related to transcriptional regulation, such as VP64 and KRAB, to coregulate gene expression, which is currently the most common use of dCas9.^59^

#### First research using CRISPR technology for disease treatment

In the months after CRISPR/Cas9 was shown to function in mammalian cells, scientists rapidly achieved gene editing in animals such as mice, fruit flies, and rats and plants such as rice and wheat.^12,60–67^ Nevertheless, treating disease was the greatest expectation of CRISPR technology, and in December 2013, Wu et al. published a study using CRISPR/Cas9 to treat cataracts in a mouse model with cataracts caused by base deletions.^68,69^ They coinjected the mRNA encoding Cas9 with an sgRNA into fertilized eggs of mice that would have cataracts, and of the 22 mouse pups obtained, ten carried the mutant allele, including six NHEJ-mediated insertions and deletions and four HDR-mediated repairs.^20–22^ All four mice with cataracts repaired by HDR induction were cured, and two of the NHEJ-induced mice were successfully cured. Based on these results, CRISPR/Cas9 can modify the genome to treat genetic diseases. In another study conducted during the same period, Schwank et al. isolated intestinal stem cells from two patients with cystic fibrosis transmembrane conductor receptor (CFTR) mutations and corrected the disease-causing mutation using CRISPR/Cas9 technology.^70–73^ In addition, they proposed a protocol for the in vitro editing of genetically mutated stem cells and their subsequent introduction into the body to treat disease, which was successfully implemented for clinical use several years later.^74^

#### Single-base gene-editing technology

Although CRISPR/Cas9 has successfully cured some diseases caused by point mutations by cleaving the double strand for re-repair, the inefficiency and uncertainty of this approach have limited its application. In 2016, Komor et al. argued that the treatment of genetic diseases should correct the mutated base rather than excising it to allow random recombination.^25,47^ Cytidine deaminase catalyzes the deamidation of cytosine into uracil, which subsequently changes back to thymine through replicative division. Thus, they integrated CRISPR/dCas9 with rat-derived cytidine deaminase (APOBEC1) and successfully achieved C to U base conversion.^75,76^ This base-editing technique was also improved to enhance the efficiency and precision of base substitution. The invention of single-base gene-editing technology not only provides a predictable method of base substitution but also designs a base substitution architecture that facilitates the subsequent invention of more base substitution methods, which is important for the treatment of genetic diseases caused by base mutations.

#### The RNA editing tool Cas13a

CRISPR/Cas13a (formerly known as C2c2) is a Class II Type VI CRISPR/Cas family protein extracted from the bacterium *Leptotrichia shahii.*^28,77^ It is characterized by the inclusion of two higher eukaryotic and prokaryotic nucleotide-binding (HEPN) domains that efficiently degrade almost all single-stranded RNAs, and the recognition of the target RNA by Cas13a is mediated by an sgRNA.^78^ Previously discovered Cas-related proteins act on the DNA strand, and the discovery of Cas13a provides a novel approach to the recognition and detection of RNA viruses, such as SARS-CoV-2.^79,80^ Cas13a has also been shown to reduce the efficiency of gene expression in a manner similar to RNAi but with greater specificity.^81^

#### First clinical trial of CRISPR/Cas9 technology

The first clinical trial of CRISPR/Cas9 technology was conducted by Lu and colleagues at West China Hospital in Sichuan, China. In October 2016, Lu et al. injected CRISPR/Cas9 gene-edited T cells back into patients, the world’s first human injection of gene-edited cells.^82^ The T cells used for gene editing were derived from patients, and plasmids encoding Cas9 and sgRNA targeting the PD-1 gene were transfected into the cells by electroporation. The data showed a significant reduction in PD-1 expression in the gene-edited T cells.^83,84^ Follow-up studies of patients who received T-cell injections showed that the patients did not experience significant adverse effects due to receiving gene-edited T cells, and two of them were in a stable condition. This study indicated the feasibility and safety of the clinical application of gene-editing technology, which is very important to promote the clinical application of gene-editing technology.

## CRISPR-based gene-editing tools

CRISPR gene-editing technology facilitates gene editing in eukaryotic cells. Researchers have studied the mechanism of action of Cas9 and have obtained Cas9 variants with different functions and some other derivative gene-editing tools through special modifications and have discovered other Cas proteins in the Cas9 family, enriching the types of genes that can be edited using CRISPR technology. Researchers have developed some vectors to assist in transport and safely deliver the CRISPR system to the body.

### Composition of CRISPR/Cas9

#### sgRNA

When invaded by exogenous phages or plasmids, bacteria and archaea containing CRISPR obtain a foreign DNA fragment inserted into the spacer region.^11^ Re-entry of the foreign nucleic acid homologous to the spacer region into the bacteria activates transcription of the CRISPR array to produce pre-crRNA. Pre-crRNA contains sequences with complementary base pairing to tracrRNA, the repeat region of the CRISPR array.^47,85^ TracrRNA first binds to the Cas9 protein after transcription; then, complementary base pairing between pre-crRNA and tracrRNA forms a double-stranded RNA, and the pre-crRNA binds to Cas9. After binding occurs, RNase III builds pre-crRNA in the primary process, and Cas9 cuts excess repetitive and spacer sequences in the secondary process.^46,86^ After the two processes, the crRNA matures and gains the ability to target the DNA strand. The backbone RNA (tracrRNA) and crRNAs that target specific sequences together comprise the sgRNA.^14,47,58^

Researchers constructed a crRNA-tracrRNA fusion transcript to simplify the aforementioned process and facilitate the application of the CRISPR/Cas9 system in eukaryotes, which greatly simplified the process of crRNA processing and maturation.^58,87^ By designing a crRNA targeting sequence of only 20 bp of bases next to the PAM site, almost any position containing the PAM site can theoretically be targeted. The major difference between CRISPR-based gene-editing technology and ZFNs and TALENs is that CRISPR-based gene-editing technology relies on the RNA-mediated recognition of the target DNA.^4,88^ The design of the sgRNA is the key to whether CRISPR gene editing is successful at the target site.

#### Cas9

The sgRNA is responsible for guiding the gene-editing system to the target site, while the modification of the target DNA strand is performed by Cas9. Using SpCas9 as an example, the binding of SpCas9 to the target DNA depends on the recognition of the PAM sequence downstream of the target site, which triggers the separation of double-stranded DNA.^89,90^ The 10 bases proximal to the PAM on crRNA are called the seed sequence, and the seed sequence first binds to the DNA strand through complementary base pairing to begin forming its R-loop structure.^91^ The distal DNA of PAM interacts with the structural domains of REC2 and REC3 of Cas9 to accelerate the formation of the R-loop, and the formation of the intact R-loop promotes the activation of the structural domains of the HNH and RuvC nucleases that catalyze the cleavage of the double-stranded DNA.^90,92–97^

When using wild-type Cas9 for gene editing, such as SpCas9 (*S. pyogenes* Cas9) and SaCas9 (*Staphylococcus aureus* Cas9), off-target effects, chromosomal translocations, large segment deletions, and other abnormalities often occur.^98–100^ Due to the limitations of the PAM, the CRISPR/Cas9 gene-editing system often fails to target the proper sites. Therefore, the modification of Cas9 focuses on two goals: enhancing the security of Cas9^101–110^ and freeing it from the limitations of PAM^111–117^ (Tables 1, 2).

**Table 1:** Cas9 variants that have been modified to broaden the scope of application

Variant name	Resources (PAM)	Selection strategy	PAM	Ref.
spCas9-NG	SpCas9 (NGG)	Elimination of the interaction between Cas9 and the third position of PAM	NG	^115^
xCas9	SpCas9	PACE	NG, GAA, GAT	^116^
Cas9-NRNH	SpCas9	PANCE and PACE that evolved by enabling SpCas9 to bind to specific sequences with non-G PAMs	NRNH	^114^
SpG	SpCas9	Structure-guided engineering	NGN	^117^
SpRY	SpCas9	Structure-guided engineering	Almost unlimited	^117^
Cas9-VQR	SpCas9	Bacterial selection system	NGAN NGCG	^341^
Cas9-EQR	SpCas9	Bacterial selection system	NGAG	^341^
SaCas9-KKH	SaCas9 (NNGRRT)	Molecular evolution and bacterial selection system	NNNRRT	^112^
eNme2-C	NmCas9 (NNNNGATT)	PANCE, ePACE, and BE-PPT	Almost unlimited	^113^
eNme2-C.NR	NmCas9	PANCE, ePACE, and BE-PPT	Almost unlimited	^113^
eNme2-T.1	NmCas9	PANCE, ePACE, and BE-PPT	NTN	^113^
eNme2-T.2	NmCas9	PANCE, ePACE, and BE-PPT	NTN	^113^

N is any nucleotide. R is A or G. H is A, C and T

*PACE* phage­assisted continuous evolution, *PANCE* phage-assisted noncontinuous evolution, *BE-PPA* base-editing-dependent PAM profiling assay, *ePACE* eVOLVER-enabled20 phage-assisted continuous evolution

**Table 2:** Cas9 variants that have been modified for increased security

Variant name	Resources	Selection strategy	Mutation domain	Ref.
SpCas9-HF1	SpCas9	Reduce the interaction between Cas9 and nontarget DNA sites	HNH and REC3 domains	^342^
eSpCas9	SpCas9	Neutralize the positive charges of Cas9 and DNA links and sites.	HNH and PAM-interacting domains	^343^
Sniper-Cas9	SpCas9	Sniper screen, an *E. coli*-based selection method		^101^
HypaCas9	SpCas9	REC3 and DNA complementation control HNH domain activation	REC3 domain	^103^
evoCas9	SpCas9	Screening method using a yeast reporter strain	REC3 domain	^102^
Cas9TX	SpCas9	Prevent the perfect repair of DNA	Carry optimized TREX2	^110^
HscCas9-v1.2	SpCas9	Substitution of amino acid residues	Multiple domains	^105^
superFi-Cas9	SpCas9	When mismatched, sgRNA, and DNA chains form RuvC loop	RuvC loop	^104^
efSaCas9	SaCas9	Construction of an SaCas9 variant library and directional screening system	REC3 domain	^106^
SaCas9-HF	SaCas9	Modify that residues where the distal region of PAM is linked to the target DNA	Recognition lobe domain and RuvC domain	^108^

### Method for CRISPR delivery

Plasmid DNA (pDNA) is an ideal vector for loading the CRISPR system because it is not easily degradable, can be amplified in large quantities, and can be easily modified.^118^ After entering the cell, the plasmid carrying CRISPR/Cas9 enters the nucleus with the assistance of NLS and transcribes the mRNA encoding Cas9 and sgRNA.^119,120^ This process is very tedious, and loading CRISPR/Cas9 tools on mRNA may greatly simplify this process. However, mRNA is easily degraded and has low stability. In particular, gene-editing tools that deliver Cas9 to function in concert with effector proteins are difficult to apply because the number of bases in the mRNA encoding Cas9 and effector proteins is too large.^121,122^

Cas9 RNPs, known as ribonucleoproteins (RNPs), are complexes formed by fusing purified Cas9 with sgRNA in vitro, and RNPs function immediately after entering cells.^123,124^ However, RNPs are relatively difficult to deliver into cells due to their complex composition and charge properties, whereas proteins and nucleic acids are usually delivered using electroporation with the assistance of cell-penetrating peptides.^125,126^ With continuous innovations in delivery vectors, scientists have identified exosomes as a promising approach to deliver Cas9 RNPs.^127,128^

### Functional categories of CRISPR tools

#### DNA strand cleavage tool

CRISPR/Cas9 was initially studied for its powerful double-stranded DNA cleavage function. The sgRNA directs Cas9 to a designated site where DSBs form flat ends in the presence of HNH and RuvC nuclease structural domains. Subsequently, DNA repair mechanisms are activated, mainly NHEJ and HDR.^21,129,130^ The repair of DSBs by NHEJ is imprecise and often leads to base mutations that result in targeted mutations. HDR repair is a complex and precise process that can repair broken DNA strands correctly. The perfectly repaired DNA strand is indistinguishable from the target DNA and will be cleaved by Cas9 again until the sgRNA becomes unrecognizable. Fortunately, the chance of HDR occurring in mature cells is much lower than that of NHEJ.^68^

Cas9 efficiently cleaves double-stranded DNA, but in practice, the sgRNA often mismatches with double-stranded DNA, leading to off-target effects.^5^ In addition, a more efficient method to mediate mutational inactivation of genes is needed to enhance the efficiency of gene knockdown and reduce unnecessary cleavage. Cas9 nickase (Cas9n), a Cas9 variant with mutations in the nuclease structural domain RuvC (D10A) of Cas9, only creates breaks in DNA strands complementary to the crRNA.^131^ DNA single-strand breaks are repaired by a high-fidelity base excision repair (BER) pathway, and thus two adjacent sgRNA/Cas9n complexes are designed to shear a single site, which effectively prevents Cas9-mediated damage to nontarget DNA and greatly enhances the specificity of Cas9.^132^ An offset of an appropriate distance between two Cas9ns facilitates the efficiency of gene editing. Zhang Feng and colleagues designed an online tool (http://www.genome-engineering.org/) for the design of two Cas9n sgRNAs to facilitate follow-up research.^131^

**Fig. 2:**
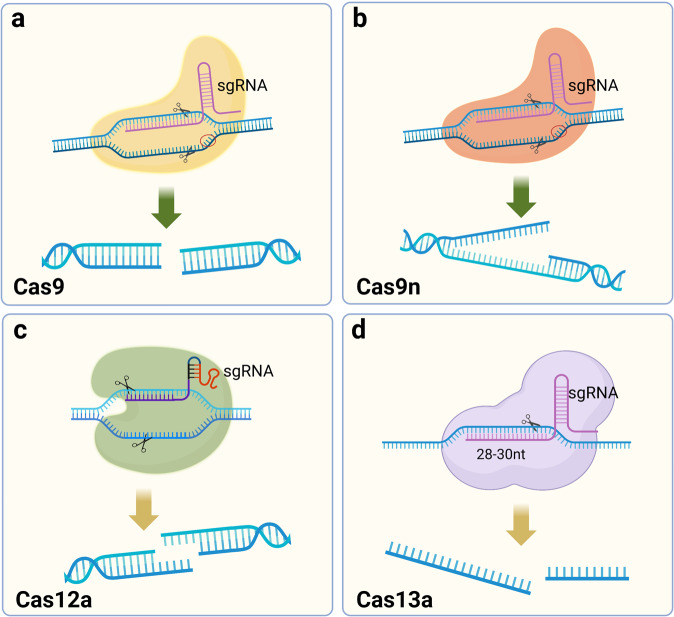
Schematic diagram of DNA strand cleavage tools. **a** Cas9 cleaves DNA double strands to form flat ends. **b** Cas9 nickase (Cas9n) cleaves the single DNA strand. **c** Cas12a cuts DNA double strands to form sticky ends. **d** Cas13a recognizes and cleaves RNA strands. (Figure was created with Biorender.com)

In 2015, Zhang Feng et al. extracted Cpf1 (CRISPR from *Prevotella* and *Francisella*), now known as Cas12a.^133^ Cas12a, belonging to the Class II Type V CRISPR‒Cas Cas12a, is a Class II Type V CRISPR‒Cas system with the same ability to cut DNA double strands as Cas9 but differs to a great extent from Cas9. In bacteria, crRNA maturation of Cas12a does not require the involvement of tracrRNA and RNase III, and when the CRISPR array is activated for transcription, the pre-crRNA is cleaved directly by Cas12a into a 43 bp nucleotide sequence serving as the sgRNA. SgRNA/Cas12a recognizes a T-rich PAM sequence, usually 5′-TTTV-3′, located upstream of the target site, followed by crRNA binding to the DNA strand. Cas12a has only one nuclease structural domain, RuvC, mediating the cleavage of double-stranded DNA.^134^ Unlike the flat ends produced by Cas9 cutting the double strand, Cas12a generates a sticky end interface similar to the double sgRNA-guided Cas9n described above, producing a 4–5 base overhang.^135^ This approach presents the advantage that if the first DNA strand repair creates insertions or deletions (indels), the target position could still be repaired the next time by HDR.^136^ The resulting sticky end interface may exert a positive effect on gene insertion in NHEJ, which must be confirmed in subsequent studies.^137,138^ In conclusion, the discovery of Cas12a enriches the gene-editing tools based on the CRISPR system, and it is the first PAM-less G-rich Cas protein that has been identified, which has important implications for some unknown genes in the genome (Fig. 2a–c).

#### Regulation of gene expression and epigenetic modification tool

The ability of CRISPR/Cas9 to cleave double-stranded DNA depends on two nuclease structural domains, and mutating both nuclease structural domains results in dCas9 that loses enzyme-mediated cleavage activity.^24^ These mutants are still able to bind to specific sites on the DNA strand under the guidance of RNA, which affects gene transcription, but more modestly, without severe off-target effects. Because Cas9 has been shown in other studies to load a number of proteins to reach a specific location in the genome and perform its function, designing a dCas9 incorporating transcription factors to regulate the expression of target genes is a potential research direction for realizing the application of the CRISPR/dCas9 system.^59^ Many diseases are often accompanied by high expression of inflammatory factors or deleterious genes during the course of development, and measures to inhibit this activation or restore the expression of protective genes are important for targeting certain chronic diseases.^139–142^ In addition, unlike CRISPR/Cas9 gene editing, because the genome has not been modified, CRISPR activation (CRISPRa) and CRISPR interference (CRISPRi) are reversible, which greatly reduces the unknown problems caused by off-target effects.^143^ Since the base sequence of DNA is not directly changed, the efficiency of gene-editing limits the application of CRISPR/dCas9.

**Fig. 3:**
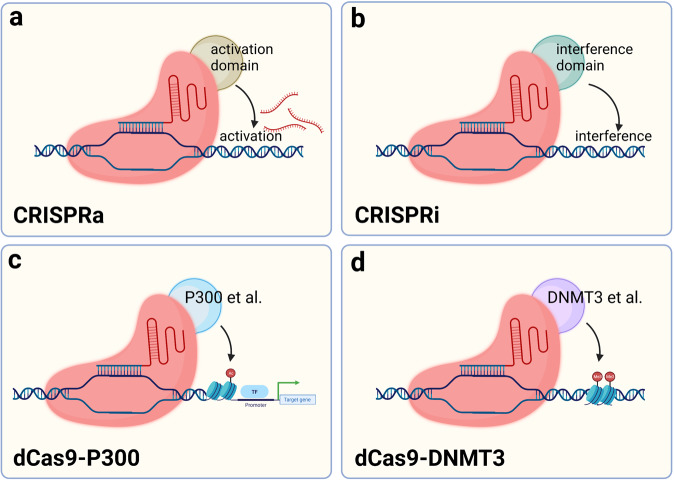
Schematic diagram of dCas9-based tools to regulate expression. **a** The dCas9 fusion VP64, VPR and other transcriptional activation effectors bind near the gene transcription start site to promote gene transcription. **b** dCas9 may be fused with KRAB or other transcriptional repressor effectors and bind to the gene transcription start site to silence gene transcription. **c** The complex formed by the fusion of dCas9 with P300 or other histone acetylases binds the gene transcription start site or enhancer region and promotes histone acetylation, which in turn enhances gene transcription. **d** dCas9 fused with DNMT3 and other DNA methyltransferases may bind the gene transcription start site to promote DNA methylation and thereby knock down gene transcription. (Figure was created with Biorender.com)

Gene regulation in eukaryotes is a complex process, and most genes are controlled by multiple regulatory elements interacting with each other. Epigenetic modifications also affect gene expression. Gilbert et al. fused dCas9 with multiple repressive chromatin modification domains and screened for a repressor domain KRAB (Krüppel-associated box) that significantly represses gene transcription.^59^ CRISPR/dCas9 binding to activating structural domains also promotes gene expression; either VP64, composed of four copies of the transcriptional activator VP16, or the p65 activating structural domain enhance transcription.^59^ A variety of activating or repressing structural domains have been developed to regulate gene expression. CRISPR/dCas9 is a universal transcriptional regulatory platform that can load activating or repressing structural domains to regulate gene expression.^144–148^ In addition, epigenetic modifications may also be regulated by dCas9 loaded with epigenetic modification enzymes such as the DNA methyltransferase DNMT3A and acetyltransferase P300.^149–151^ The risk of off-target effects of CRISPR/dCas9 is much lower than that of Cas9, and the effect is relatively efficient and mild, but the mechanism regulating gene expression is very complex. Thus, designing an sgRNA that targets one site may result in altered expression of multiple genes, and the risks must be further explored by performing more in-depth studies^152^ (Fig. 3).

#### Base-editing technology

Many known genetic diseases are caused by a mutation in a base in a gene. The fundamental aim in treating these diseases is to restore the mutated base to the original base, not to cleave the DNA strand so that random repair mediated by HDR or NHEJ occurs, and the existing gene-editing tools are unable to achieve the desired function.^23^ The five nucleotides are structurally similar to each other; for example, cytidine deaminase catalyzes the deamination of C into U. In the nucleus, U is replaced with T during cytokinesis, resulting in a C-G to T-A substitution, by agents later classified as cytosine base editors (CBEs).^153–155^

Komor et al. selected APOBEC1 from rats as a first-generation base-editing tool (BE1) by comparing cytidine deaminase activity from humans, rats, and lamprey.^25^ Catalytically inactive dCas9 was chosen as the target delivery vehicle to carry catalase, and 16-residue XTEN was also added as a stabilizer for this system. BE1 has good deamidation activity against nucleotides at the distal 4–8 positions of PAM, but in human genomic experiments, the conversion efficiency was only 0.8–7.7%. A possible explanation is that U is a base that does not belong in DNA and is easily repaired during DNA repair. In the second generation of base-editing tools (BE2), a stabilizer for U was added to prevent BER.^156,157^ This improvement was successful, achieving a three-fold increase in the base substitution efficiency of BE2. Catalyzing a strand break complementary to the mutation site to replace G with A when BER occurs further improves the efficiency of base substitution.^24,131^ Subsequent researchers have made some improvements to BE3 to enhance the efficiency of base editing, such as modifying and optimizing the nuclear localization signal, changing codons, and other methods, to improve the efficiency of BE3 base editing, reduce the formation of indels, and obtain more efficient gene-editing tools such as BE4max and BE4-Gam.^158–160^ Kurt et al. successfully developed a gene-editing tool to induce a C-to-G substitution based on BE4max.^76^ CBE frequently undergoes C-G mismutations during the process of achieving the C-to-T substitution, and the addition of two UGIs effectively stops this process. The human UNG (hUNG) enzyme with increased abasic site generation also has positive implications for base replacement between C and G.^160^ Through a series of improvements, a novel base editor (BE4max (R33A) ΔUGI-hUNG complex (CGBE1)) was finally obtained.^76^ This study improved the gene-editing tools for interbase substitution and facilitated the development of C-to-G base editors.

Achieving C-G to T-A and C-G to G-C substitutions is important for single-gene-editing efforts, but multiple types of base mutations cause disease, and achieving arbitrary substitutions between bases is an urgent task for applying CRISPR technology to disease treatment.^161^ In 2017, Liu and his colleagues completed work to replace A-T base pairs with C-G base pairs.^26^ Unlike the C-to-U substitution, which has been reported to occur only on free adenine, adenosine in RNA or adenosine in RNA‒DNA mismatches, no adenine deaminases are capable of deaminating A on double-stranded DNA. TadA is a tRNA adenine deaminase, and because of its homology to APOBECs, modifying TadA so that it can activate adenine deaminase activity on the DNA double strand is a promising approach.^162,163^ When the antibiotic resistance gene in *E. coli* was mutated, *E. coli* survived only if they obtained the mutant site to achieve an A-to-I substitution. Using this method, researchers screened for TadA* capable of acting as a mutation on the DNA strand. During *E. coli* selection, the survival rate of *E. coli* in the presence of heterodimeric TadA-TadA* was higher, and the formation of heterodimers might significantly improve the editing efficiency of adenine bases. TadA-TadA*-Cas9n was finalized as ABE7.10 through several modifications.^152^ Adenine is catalyzed by adenosine deaminase to become inosine, which eventually leads to the conversion of A-T to G-C. In subsequent studies of ABE, additional improvements were made to ABE7.10 to obtain a more efficient base-editing tool with fewer side effects.^159,164–166^

The cytidine deaminase AID from a human source fused to the C-terminus of nCas9 efficiently achieves C-to-T editing.^167^ However, in some strains, researchers have detected a high frequency of C-to-A mutations. CBEs added UGI to suppress the activity of the uracil-DNA glycosylase (*ung*) gene to increase the frequency of C-to-T mutations.^25,168^ A high frequency of C-to-A mutations was observed in strains without suppressed *ung* activity, and this gene may be responsible for the C-to-A mutation. Finally, the Ung-nCas9-AID complex was constructed. This complex enables efficient C-to-A base substitution and fills a gap in single-base gene-editing technology.^75^ Similarly, *ung* genes are involved in C-to-G base substitutions, and they construct the APOBEC-nCas9-Ung complex that allows efficient C-to-G substitutions. Researchers refer to this nCas9-cytidine deaminase-*ung* substitution as glycosylase base editors (GBEs).

**Fig. 4:**
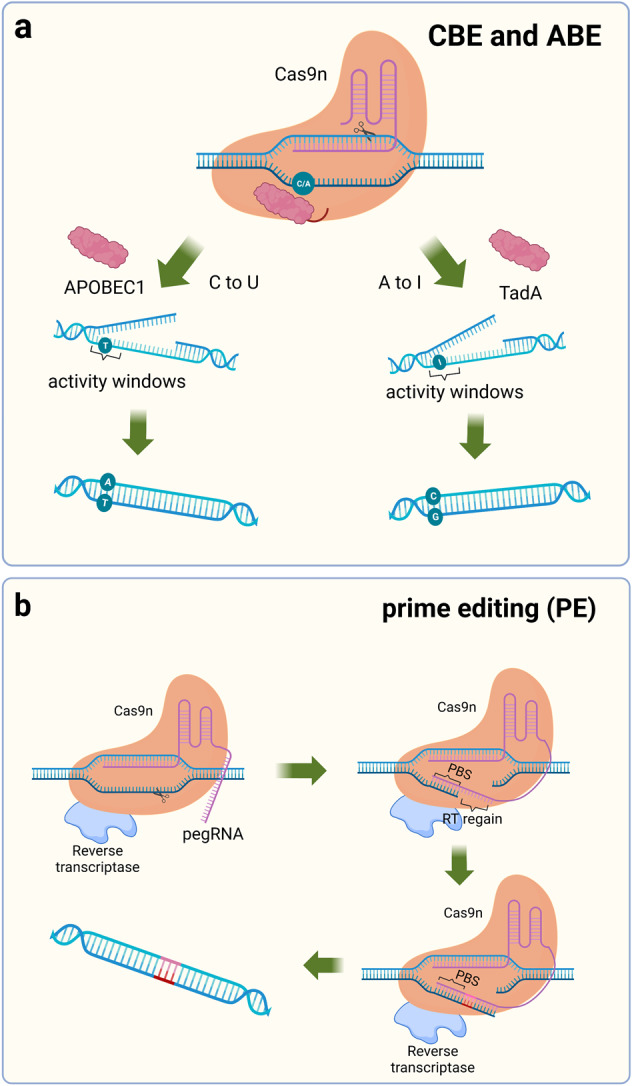
Schematic diagram of the single-base substitution tool. **a** Fusion of Cas9n with adenosine deaminase or cytidine deaminase enables the introduction of point mutations in the genome, APOBEC1 induces a C to U mutation, and TadA induces an A to I mutation. **b** PE contains a 30 bp segment of pegRNA, including the PBS sequence and RT region. PBS binds to the DNA strand and synthesizes the complementary strand of the RT region in the presence of reverse transcriptase. PBS primer binding site, RT reverse transcriptase, pegRNA prime editing guide RNA. (Figure was created with Biorender.com)

Both ABE and CBE show efficient base substitution but do not achieve insertions, substitutions, and deletions between bases at will. Thus, a new single-base-editing technology may be needed.^169^ The prime editor (PE) consists of two parts, a reverse transcriptase (RT) protein from *Moloney murine leukemia* virus (M-MLV) fused with Cas9n (H840A) and a 30 bp sgRNA (pegRNA), including a primer binding site (PBS) and an RT template.^27,170–173^ After Cas9n reaches the designated position, it cuts the target DNA strand. PBS fixes the free 3′ DNA strand by complementary base pairing and reverse transcribes the new DNA strand with the RT template under the action of RT. Using this approach, arbitrary substitutions between bases are achieved, greatly increasing the applicability range of single-base gene editing, and base insertions and deletions can also be introduced. PE2 was obtained by optimizing M-MLV RT based on PE1, and the bases on the unedited strand must rely on DNA repair mechanisms to change.^27^ BE3 in the system described above was modified by shearing the nonedited strand to obtain a much higher mutation efficiency than BE2. Therefore, in the improved PE2, another new sgRNA was added to cleave the nonedited strand to obtain PE3 and PE3b.^27^ Although the editing efficiency of PE3/PE3b was increased by ~3-fold, Cas9 was unable to discriminate between these two different sgRNAs, introducing an unknown risk for this editing system (Fig. 4).

#### Tools for RNA strand identification and cleavage

CRISPR/Cas13a is an acquired immune defense mechanism for bacteria against RNA infestation.^28^ Unlike Cas9, Cas13a recognizes the RNA strand and cleaves it using the HEPN nuclease structural domain. After cleaving the target RNA, the RNase activity is retained. The specificity is significantly reduced, leading to the cleavage of other nontarget RNAs, a phenomenon called collateral cleavage. RNA has an important role in the cell, and a relatively simple way to knockdown RNA has been developed based on a gene function screen. RNAi has good knockdown efficiency, but off-target effects are difficult to avoid.^174,175^ CRISPR/Cas13a-based RNA gene-editing tools play a comparable role to RNAi but with much lower off-target efficiency.^81^ Catalytically inactivated dCas13a, similar to dCas9, carries the corresponding effectors to regulate the function or translation of RNA, such as by regulating widespread m6A methylation on RNA, modifying the bases of RNA, and regulating protein translation^176–181^ (Fig. 2d).

CRISPR/Cas13a is also widely used to detect RNA.^78^ In 2017, Zhang Feng and colleagues designed a nucleic acid detection tool called Specific High Sensitivity Enzymatic Reporter UnLOCKing (SHERLOCK) based on Cas13a.^182^ They designed a reporter molecule that releases a fluorescent signal when the target single-stranded RNA (ssRNA) breaks, and they coincubated the constructed Cas13a, reporter molecule and crRNA with the target ssRNA and successfully observed the fluorescence; however, this approach is less sensitive. The amount of ssRNA detectable by Cas13a was increased by amplifying RNA using recombinase polymerase amplification (RPA) and T7 transcript binding to improve the sensitivity of Cas13a-based detection.^183^ This method was improved for SARS-CoV-2 detection in 2020.^184^

### Carriers for delivering CRISPR technology

**Fig. 5:**
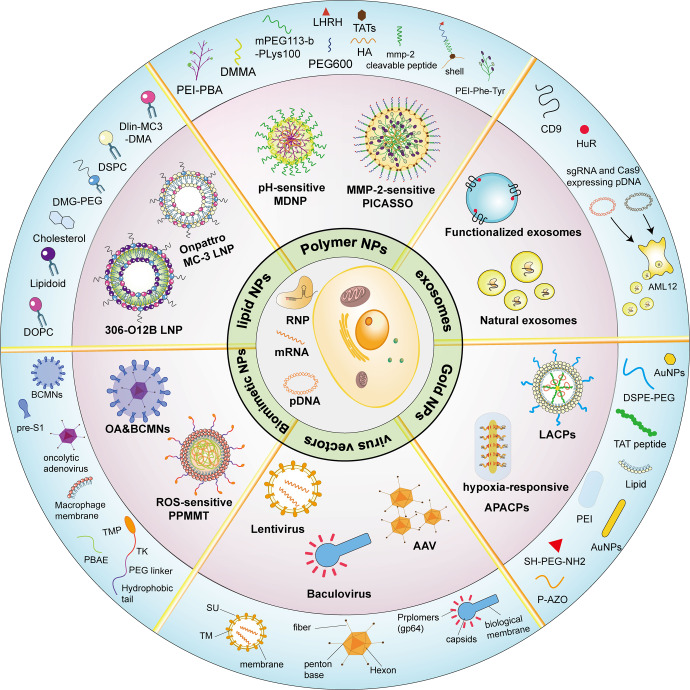
Schematic diagram showing multiple types of vectors for the in vivo delivery of CRISPR systems. The central region shows three forms of CRISPR action: pDNA, mRNA, and RNP. The middle circle section shows examples of delivery carriers, and the outermost area shows how the carriers are produced or the components. SU surface envelope protein, TM transmembrane envelope protein. (Figure was created with Adobe Illustrator and Biorender.com)

Plasmids or mRNAs loaded with CRISPR/Cas gene-editing systems are transfected into cells in vitro in the same manner as ordinary nucleic acids using transfection reagents, virus-mediated transfection, and other techniques. RNPs also enter cells through electroporation. However, most of these methods are less suitable in animals or humans. CRISPR tools undergo a long delivery process composed of three main phases to be effective in vivo: (1) the carrier must remain stable in the blood without degradation or immune clearance, (2) the carrier then accumulates in candidate tissues and triggers cell endocytosis, and (3) the CRISPR system escapes the lysosome into the cytoplasm to perform genome editing or regulate gene expression, particularly in the second phase of delivery, where enrichment in the target tissue is critical for successful delivery. The realization of this complex process requires the help of several delivery vehicles (Fig. 5).

#### Virus vectors

In previous studies, viral vectors have been commonly used to deliver gene drugs. AAV is one of the most commonly used viral vectors for delivery, as it easily crosses the species barrier to infect cells and has very low immunogenicity, making it less likely to trigger an inflammatory response.^30^ However, the CRISPR/Cas9 gene-editing system is very large compared to ordinary gene drugs, exceeding the maximum packaging capacity of AAV vectors by 4.7 kb.^185^ In particular, when Cas9 carries effector proteins, special modifications are required for loading in AAV vectors, such as using the smaller SaCas9 or splitting the delivery system into two vectors.^186–188^ Incorporating the coding sequence of the smaller Cas9 ortholog, SaCas9, into the regulatory cassette allows the coinclusion of effector-encoding sequences as epigenetic regulators to facilitate Cas9 regulatory activity while maintaining the plasmid size within the carrying capacity of AAV. For example, Himeda et al. established a CRISPRi system with dead SaCas9 (dSaCas9) and successfully inhibited the expression of full-length *DUX4* mRNA (DUX-fl) in vitro, alleviating facioscapulohumeral muscular dystrophy (FSHD).^139^ Double AAV vectors incorporate separately designed plasmids encoding a split Cas9 to accommodate the limited AAV carrying capacity, along with sgRNA. Upon cotransfection into a cell, the full Cas9 protein and sgRNA are produced to modulate gene expression; however, this method has a high risk of off-target effects.

Lentiviruses are retroviruses that infect dividing and nondividing cells and are therefore also often used as delivery vectors.^189^ Due to the 10 kb loading capacity of lentiviruses, the entire CRISPR/Cas9 system can be loaded into it, but because lentiviruses integrate randomly into the host genome, they often trigger some immune responses and even cause cancer.^29^ Baculovirus has also been used for CRISPR/Cas9 delivery. Baculovirus is a nonpathogenic insect virus with an extra-large loading capacity (~38 kb).^190,191^ Moreover, as these viruses neither duplicate nor integrate into the genome, they have no heritability concerns. Nguyen et al. engineered baculovirus as a dCas9-VP64-p65-Rta (dCas9/VPR) delivery vehicle to significantly activate endogenous long noncoding RNA (lncRNA) differentiation-antagonizing nonprotein coding RNA (DANCR) in bone marrow-derived mesenchymal stem cells (BMSCs) and rat adipose-derived stem cells (rASCs).^192^

#### Lipid-based nanocarriers

The first use of cationic liposomes for DNA transfection was reported in 1987 when Felgner et al. discovered the ability to use liposomes for gene delivery.^193^ In the following decades, liposomes were frequently used as vectors for gene drug delivery, and liposome-based nanoparticles (LNPs) are considered promising tools for CRISPR/Cas9 transfer.^29^ Unlike liposomes, LNPs do not have a continuous lipid bilayer and large inner aqueous pool, but they are mainly composed of lipid components such as natural phospholipids, cholesterol, and polyethylene glycol.^32^ The simple synthesis of LNPs and their stable presence in serum have led to their frequent adaptation for the in vivo delivery of gene drugs. Unfortunately, since the liver is the dominant organ metabolizing lipids, lipid nanoparticles always show a high degree of enrichment in the liver. This targeting is very beneficial for the delivery of drugs for the treatment of liver diseases, but LNPs do not show high efficiency for diseases occurring in other organs.^194^

Angiopoietin-like 3 (Angptl3) is an enzyme that regulates plasma lipoprotein levels. Loss of Angptl3 function reduces blood levels of triglycerides (TGs) and low-density lipoprotein cholesterol (LDL-C) without causing any clinical risk. Qiu et al. designed multiple LNPs for the delivery of Cas9 mRNA and an sgRNA targeting Angptl3.^195,196^ A gold standard MC-3 LNP configured with cholesterol, DSPC and DMG-PEG was used as a control to screen for the most efficient 306-O12B LNP consisting of a leading tail-branched bioreducible lipidoid (306-O12B) and an optimized mixture of excipient lipid molecules.^197^ The gene-editing efficiency of this LNP reached 38.5%, which is ~12 times that of MC-3 LNP.^195^ The modification of LNPs to increase their enrichment in extrahepatic tissues might improve the scope of application of LNPs to deliver CRISPR/Cas9 for disease treatment. For example, Mohanna et al. constructed the novel LNP-based Incisive Delivery System (DS) that detected extensive genome editing in mouse corneas,^198^ and Rosenblum et al. designed CRISPR-LNPs (sgPLK1-cLNPs) for tumor cells and observed ~80% gene-editing efficiency in tumor cells in vivo.^199^

#### Polymer-based nanoparticles

Polymer nanoparticles have the advantages of low immunogenicity, good biocompatibility, and a high modification potential.^200^ PLGA, chitosan, and other molecules, which are commonly used to construct polymer nanoparticle shells, improve the efficiency of polymer uptake by cells. The PEI of the core is often used as a transfection reagent for plasmid transfection, which is endocytosed by the cell and triggers the proton sponge effect into the cytoplasm.^201^ In addition, polypeptides that recognize cell membrane surface receptors and polymers that are released by catabolism at specific pH, ATP, and hydrogen peroxide levels have be designed on polymer-based nanoparticle shells.^33,202–205^

Liu et al. constructed a multistage delivery nanoparticle (MDNP) for delivering the CRISPR-dCas9 system.^206^ They built the core-shell structure. The cationic polymer formed by PEI nanoparticles modified by phenylboronic acid (PBA) was used as the core. This core was then fused to the plasmid encoding dCas9 and sgRNA. The use of 2,3-dimethylmaleic anhydride (DMMA)-modified poly(ethylene glycol)-b-12 polylysine (mPEG113-b-PLys100/DMMA) as a shell wrapping the abovementioned cationic polymer allows the nanoparticles to exhibit different surface properties at different stages. The nanoparticles are injected into the bloodstream through the tail vein and stabilize in the bloodstream due to the negatively charged PEGylated surface of the shell. The tumor tissue has an acidic microenvironment (pH 6.5) in which the polymer shell rapidly dissociates and the core of the polymer becomes exposed due to a high level of surface sialylation on the surface of the cancer cells. The PBA moiety of the core binds to sialic acid, enhancing endocytosis by tumor cells. In cancer cells, PEI in the nucleus of the multimeric body escapes from lysosome via the proton sponge effect, causing water molecules and chloride ions from the lysosome to flow inward and plasmid DNA (pDNA) to successfully enter the cytoplasm of cancer cells. MDNPs overcome physiological barriers by changing the surface chemistry several times before finally entering tumor cells effectively. Changing the plasmids loaded with MDNP should allow it to become a novel technology for cancer treatment.

A dual-locking nanoparticle (DLNP) is another polymeric particle reported from the same team who developed MDNP.^207^ DLNPs have a CRISPR/Cas13a core that targets PD-L1 in tumor cells. Cas13a enters tumor cells and is activated upon specific recognition of the PD-L1 mRNA. Activated Cas13a nonspecifically cleaves RNA and triggers the apoptosis of tumor cells. The tumor microenvironment has many typical features, and the slightly acidic environment may serve as a marker for polymer-based nanoparticles to discriminate tumors. In addition, reactive oxygen species (ROS) are also present at higher levels in the tumor environment than in normal tissues, and ROS also promote cellular DNA mutation and tumorigenesis.^208^ The authors designed a responsive shell that disintegrates only under specific ROS and pH conditions to minimize irreversible damage to cells in other organs due to DLNP off-targeting. After DLNPs enter the body through the bloodstream, they are protected from immune clearance due to the presence of polyethylene glycol on their surface. When DLNPs reach the tumor through the blood, the microacidic environment and high ROS concentration in the tumor drive the disintegration of the DLNP shell, exposing the polymer core of the PEI/Cas13a complex. Eventually, the core is internalized into the tumor cells and released into the cytoplasm through the proton sponge effect.

The application of the CRISPR/dCas9 system in regenerative medicine is a hot topic. A layer-by-layer self-assembled peptide (SAP) coating was prepared on nanofibers and used to deliver the CRISPR-dCas9 system to promote the neurite growth of rat neurons.^209^ Polycaprolactone (PCL) has several advantages that make it ideal for delivering the CRISPR-dCas9 system, including good stability, easy processing, good biocompatibility, and the ability to biodegrade.^210^ However, experiments inspired by mussel adhesion chemistry showed that PCL does not readily adhere to cells. Zhang et al. developed a new method for PCL attachment using a layer of negatively charged amphiphilic SAP, and the pDNA encoding the CRISPRa system and SAP-RGD was absorbed through electrostatic interactions.^209^ The RGD polymorphism supported cell adhesion and proliferation, effectively resolving the deficiency in PCL adhesion. SAP has a good affinity for many other biological peptides, and attaching SAP coatings to PCL is expected to be a routine strategy employed for in vivo targeted delivery of CRISPR/Cas9.

#### Natural and functionalized exosomes

Exosomes are membrane-bound vesicles that are 30–100 nm in diameter and originate from multivesicular bodies (MVBs) in organelles.^211,212^ In living organisms, exosomes serve as a medium for intercellular transfer of proteins, lipids, nucleic acids, and other intracellular factors and carry virtually any biological component, including plasmids, with minimal side effects.^213,214^ As exosomes also directly package sgRNAs and Cas9, thereby effectively decreasing the risk of off-target side effects during transport, they constitute a promising vehicle for CRISPR/dCas9 system delivery. Moreover, because exosomes retain proteins and lipids reflecting those of the parent cells, they preferentially interact and fuse with the parent cell type.^215,216^

Hepatic stellate cells (HSCs) secrete a large number of exosomes, and the exosomes secreted by these immortalized cells are less different from each other and more workable.^128^ RNP is packaged in exosomes by electroporation to obtain the genome editing system exosome^RNP^. Wan et al. designed sgRNAs targeting p53 upregulated modulator of apoptosis (PUMA), Cyclin E1 (CcnE1), and K (lysine) acetyltransferase 5 (KAT5), which play important roles in liver disease development, in combination with Cas9 to construct the RNP.^217–219^ A significant decrease in the expression of all three genes was detected. Exosome^RNP^ is highly enriched in the liver and is an ideal vehicle for the targeted treatment of liver diseases such as cirrhosis and liver fibrosis. Nevertheless, artificial modifications are needed to enhance the exosome carrier targeting ability for certain cell types with low exosome secretion. Genome editing with designed extracellular vesicles (GEDEX) was developed for dCas9/VPR delivery to increase the delivery efficiency and precision.^220^ Conversion of hepatic stellate cells (HSCs) to myofibroblasts (MFBs) is an important marker of liver fibrosis formation.^221^ Upregulation of growth factor expression in endogenous hepatocytes effectively repairs liver injury in mice. GEDEX is similar to naturally occurring exosomes and thus can be modified to target a wide range of cells in vivo, highlighting its considerable potential for future clinical application.

Li et al. constructed a novel exosome by fusing the CD9 C-terminus with human antigen R (HuR) to improve the encapsulation ability of exosomes.^222^ The length of the dCas9 mRNA increases the difficulty of encapsulating molecules in exosomes using methods such as electroporation, and the HuR recognition motif on this novel exosome facilitates dCas9 loading and thus shows significant promise for the targeted delivery of CRISPR/dCas9 systems to treat diseases.

Exosomes are endogenous delivery vehicles. They are less impeded due to their compositional similarities to cell membranes and are therefore less likely to be cleared by the immune system during cargo delivery than viral vectors, lipid nanocarriers, and polymorphic nanocarriers.

#### Gold nanoparticle delivery systems

Gold nanoparticles can be customized in size, and different sizes have different physical and chemical properties. One of the most typical features is that the surface electrons of Au nanoparticles (AuNPs) resonate at a frequency determined by the size of the NP, a phenomenon known in the scientific community as surface plasmon resonance.^223,224^ Surface plasmon resonance is the most important application of AuNPs, which are used to prepare various low-cost sensors that can be observed with the naked eye.^225,226^ Moreover, gold nanoparticles have good stability and biocompatibility, and surface modifications can easily be added, which makes them ideal carriers for delivering gene drugs.^227^ In the treatment of diseases such as tumors, for example, the surface of AuNPs may be decorated with specific cancer cell ligands to enhance their recognition of cancerous tissue. In addition, gold nanoparticles themselves have anti-inflammatory and antibacterial properties, which are beneficial in the treatment of tumors.

Wang et al. combined lipid nanoparticles with good stability and a high drug loading rate with AuNPs that were released in vitro in a controlled manner.^228^ A lipid-encapsulated AuNP/Cas9-sgPlk-1 plasmid (LACP) that was targeted for delivery to melanoma-bearing sites was constructed. First, the authors prepared AuNPs with a diameter of ~20 nm, which were attached to the TAT peptide, increasing the uptake of NPs by cells.^229^ AuNPs form the core of the polymer through electrostatic interactions with negatively charged pDNA, and finally, the core is wrapped with cationic liposomes and then modified with PEG2000-DSPE to form LACP. The lipid shell stabilizes the structure of LACP and enhances cellular internalization. TAT directs nuclear targeting, and the AuNP core acts as both a carrier and a responder to photothermal conditions to release pDNA.

#### Biomimetic nanomaterials

The stable presence of nanomaterials in the circulation and their ability to undergo enriched accumulation at specific sites in the body can enhance the therapeutic efficacy of CRISPR gene-editing drugs.^230^ However, carefully designed organic or inorganic carriers are inevitably partially cleared by the immune system in vivo. Researchers have expressed widespread interest in the use of a material from the organism itself, a biofilm that serves as a “pocket” for the contents of the cellular envelope, to prevent recognition by the immune system.^231^ When a disease occurs, immune cells are usually triggered to enter the disease site and exert anti-inflammatory effects. The encapsulation of nanomaterials in the cell membranes of these cells not only prevents possible immune clearance but also enhances the enrichment of gene drugs at the disease site.^232^

Yan et al. used the cationic polymer poly (β-amino ester) (PBAE) in complex with a plasmid encoding the CRISPR system as the core and covered the surface of the PBAE/pDNA complex with a macrophage membrane. Finally, the ROS response element (BAM-TK-TMP) was fused to the outer surface of the cell membrane.^233^ In this bionanomaterial, the macrophage membrane targets inflammatory lesions, and TMP recognizes high ROS levels to promote the cellular internalization of nanoparticles. TMP may be tailored as an effector in response to multiple pathological or physiological conditions.^234–236^

The occurrence of disease in vivo involves multiple genes, biochemical properties, and changes in the microenvironment. This complex mechanism poses great difficulties to drug delivery carriers, and LNPs, gold nanoparticles and bionanomaterials each have their own advantages and limitations. Moreover, various nanocarriers can be connected together, and the construction of composite nanoparticles can employ the different advantages of the carriers and enhance the delivery efficiency.^237^ For example, Zhang et al. fused poly(ethylene glycol) methyl ether-block-poly(lactide-co-glycolide) (PEG-b-PLGA;PP)-based nanoparticles with PEI to obtain composite lipid and polymer nanoparticles: PP/PEI.^238^ PP/PEI prevents the increased enrichment of lipid nanoparticles in the liver and allows efficient genome editing in the lung, heart, and blood vessels of adult mice after the administration of a single dose. In a follow-up study, the researchers also found that polyethylene glycolized nanoparticles have the ability to inhibit gene drug aggregation in individual organs and increase the duration of circulation in the body. In addition, the aforementioned delivery vehicle, LACP, is also a composite nanoparticle of liposomes and AuNPs.^228^ In conclusion, the rational use of the advantages of various nanoparticles to design nanocarrier structures facilitates the combination of several excellent platforms for delivering gene drugs individually, which improves the efficiency of gene drug delivery and helps optimize the therapeutic effects of gene drugs (Table 3).

**Table 3:** Characteristics of various vectors loaded with the CRISPR/dCas9 system

Delivery vector	Advantages of delivering CRISPR systems	Disadvantages of delivering CRISPR systems	Ref.
AAV vector	High delivery efficiency, security, clinical certification	Limited packaging capacity, high cost, pathogenicity	^139,188^
Lentivirus vector	High delivery efficiency, low immunogenicity	High cost, construction is difficult	^189^
Baculovirus vector	Large packaging capacity, low genotoxicity	Limited delivery efficiency	^344^
Lipid-based nanoparticles	Easy to transform, security, large packaging capacity	Easily ingested by the liver	^345^
Polymer-based nanoparticles	Easy to transform, controllable release	Complicated packaging construct, limited application	^86^
Natural exosomes	High biocompatibility, easy access	Easily ingested by the liver	^346^
Functional exosomes	High biocompatibility, high specificity	Medium delivery efficiency, easily ingested by the liver	^346^
AuNPs	Easy to transform, anti-inflammatory properties, special physicochemical properties, large packaging capacity	High cost	^347^
Biomimetic nanoparticles	High biocompatibility, easy to transform, anti-inflammatory properties, large packaging capacity	Limited delivery efficiency	^348^

## Application of gene-editing tools

In the preceding sections, we summarized a variety of genome-altering gene-editing approaches involving CRISPR systems and summarized the vectors available for delivering CRISPR tools in vivo or in vitro, including some brief descriptions of the characteristics, improvement options and applicability of these vectors. Next, we analyzed the alteration of the disease microenvironment or the salient features of diseased cells at the onset of some diseases from the disease perspective. We summarize the most promising CRISPR gene-editing tools with targeted delivery vectors for different types of diseases to facilitate subsequent studies.

### Attempts to treat diseases

#### Cancer

**Fig. 6:**
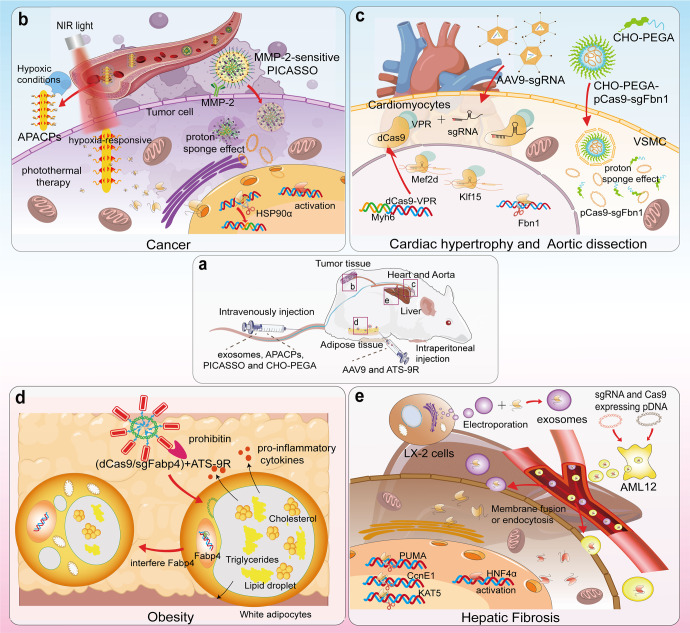
Delivering the CRISPR/Cas9 system to treat cancer, liver fibrosis, obesity, and cardiovascular diseases. **a** APACPs, exosomes, PICASSO and CHO-PEGA were intravenously injected into mice, and AAV9 was intraperitoneally injected. **b** APACPs are transported through the blood circulation to the tumor tissue, and hypoxic conditions promote the entry of APACPs into tumor cells. NPs release RNPs, silence the expression of HSP90α and reduce the hyperthermia tolerance of tumor cells. Externally applied NIR-induced photothermal therapy kills tumor cells. PICASSO responds to MMP-2 on the tumor cell membrane, and the shell disintegrates and the core enters the cell by endocytosis. The plasmid escapes from the lysosome into the cytoplasm through the proton sponge effect. **c** AAV9 delivered sgRNAs targeting Mef2d and Klf15 into dCas9-VPR transgenic mice. dCas9-VPR was synergistically transcribed with Myh6 and therefore specifically activated the expression of Mef2d and Klf15 in cardiomyocytes. Lipid nanoparticles CHO-PEGA deliver CRISPR/Cas9 to vascular smooth muscle cells in aortic coarctation to knockdown Fbn1. **d** Adipocyte targeting sequence to 9-mer arginine (ATS-9R) recognizes forbidden elements expressed at high levels in adipose tissue and delivers plasmids into white adipocytes, which contain huge lipid droplets and large amounts of triglycerides and cholesterol and release large amounts of inflammatory factors. After interfering with the expression of fatty acid binding protein 4 (Fabp4), the size of lipid droplets in white adipocytes decreases, and the release of inflammatory factors is inhibited for the purpose of treating obesity. **e** The exosomes secreted by LX-2 cells were extracted, and RNPs were loaded into the exosomes by electroporation. In studies targeting the knockdown of PUMA, CcnE1, and KAT5, exosomes were effective at alleviating liver diseases such as liver fibrosis. In vitro transfection of plasmids encoding sgRNA and dCas9/VP64 into mouse liver AML12 cells resulted in the secretion of AML12 exosomes carrying the CRISPR/dCas9 system. Delivery of these exosomes to HSCs elevated HNF4α expression and prompted cell differentiation into hepatocytes. (Figure was created with Adobe Illustrator and Biorender.com)

Cancer, a disease with high incidence and mortality rates, is standardly treated using surgical resection, radiotherapy, and chemotherapy; however, the latter two treatments engender serious side effects.^239^ The process of cancer development is usually accompanied by abnormal expression of large numbers of genes, such as P53, Notch, and PD-L1.^240–243^ In addition, the microenvironment in which tumorigenesis occurs also exhibits some abnormal changes. These characteristics are used to construct a nanoparticle that is released in a specific environment and efficiently deliver gene drugs to tumor cells. In tumor cells, aberrantly expressed genes may be silenced or overexpressed using CRISPR technology (Fig. 6b).

Rapid cell proliferation is a distinct feature of cancer development, a process that requires high oxygen consumption, leaving the tumor in a hypoxic microenvironment.^244^ Photothermal therapy is defined as the delivery of infrared light-responsive nanomaterials to the body and the external application of infrared light to produce localized heat in the body and ablate tumors.^245^ However, tumors can tolerate high temperatures up to 50 °C, which may lead to damage to paracancerous tissue. Therefore, reducing the temperature tolerance of tumor cells and using photothermal therapy at moderate to low temperatures may effectively hamper tumor development. The protein heat shock protein 90α (HSP90α), which is associated with cellular heat tolerance and is overexpressed in tumor cells, was obtained in a screen. Li et al. constructed a hypoxia-responsive nanoparticle based on gold nanorods that carried a CRISPR/Cas9 system to target HSP90α for knockdown.^244^ After the vector entered the tumor cells, Cas9/sgRNA RNP was released into the cells, silencing HSP90α and causing the cells to lose their thermotolerance. Finally, infrared light activated the gold nanorods to ablate the tumor.

T lymphocytes play an important role in cancer immunotherapy.^246^ The T-cell receptor (TCR) on the surface of the T-cell membrane recognizes peptides or antigens that bind to MHC molecules to identify and kill tumor cells.^247^ For example, the surface of myeloma, melanoma and sarcoma cells contains NY-ESO-1 as an antigen that binds the MHC molecule.^248–250^ However, mismatches between the therapeutic TCRα and β chains in T cells and endogenous TCR chains (TRAC and TRAB) reduce the expression of surface TCRs.^251,252^ In addition, one study found that T cells from PD-1-deficient mice were more potent against cancer.^253^ CRISPR was then used to knock out genes encoding TRAC, TRAB, and PD-1 (such as PDCD1) in vitro to improve the safety and efficacy of engineered T cells. This therapeutic strategy, called TCR-T therapy, significantly inhibited the growth of both hematologic and solid tumors. In addition, another study modified T cells to treat tumors, called CAR-T cells.^254^ Tumor cells are killed by inserting a CAR gene-targeting CD19 into T cells, but the experiments have been indicated to be ineffective against solid tumors. Both TCR-T and CAR-T cells have been approved by the FDA and have been proven to be effective and safe in clinical trials. Other characteristics of tumor tissue or cells also have the potential to be applied for targeted drug recognition, such as membrane surface receptors, the tumor microenvironment, and proteins undergoing specific modifications.^255,256^

#### Liver diseases

The liver is the main organ in the body that metabolizes lipids, and liposomes and lipid nanoparticles without special modifications are more likely to be enriched in the liver.^257–259^ In addition, many cells in the liver secrete large amounts of exosomes, and these hepatocyte-derived exosomes with a homologous tissue targeting ability will be more easily enriched in the liver.^260^ In contrast to synthetic lipid nanoparticles, these naturally occurring exosomes are originally carriers for the intercellular delivery of proteins, nucleic acids, and other molecules. Therefore, natural exosomes are very safe and are rarely cleared by the immune system (Fig. 6e).

Various cell types in the liver produce or take up exosomes.^261^ Thus, as exosomes from HSCs participate in establishing liver fibrosis, hepatocyte-derived exosomes may carry therapeutics for delivery into HSCs. Endogenous liver exosomes derived from the mouse liver AML12 cell line are thus safer and more effective as vectors. Encapsulation of the CRISPR-dCas9-VP64 system into AML12-derived exosomes successfully activated the expression of hepatocyte nuclear factor 4α (HNF4α), a transcriptional regulator of hepatocyte differentiation, in HSCs and a mouse model of liver fibrosis, thereby significantly attenuating liver fibrosis. In another experiment for the treatment of liver fibrosis, Luo et al. transfected plasmids expressing dCas/VP64 and sgRNA into the mouse hepatocyte line AML12.^261^ The presence of dCas9 was detected in exosomes, suggesting that RNPs with transcriptional activity can be loaded in exosomes.

Phenylketonuria (PKU) is an autosomal recessive liver disease in which phenylalanine hydroxylase (PAH) enzyme deficiency results in decreased phenylalanine metabolism, causing hyperphenylalaninemia.^262^ Repair of mutated bases using a single-base editor that converts C-G base pairs to T-A base pairs restores PAH expression and increases the reduced level of phenylalanine in blood. Villiger et al. used AAV carrying a single-base gene editor delivered to the Pah^enu2^ mouse model, and 63% of the mRNA had the corrected base sequence, a result that confirmed the effectiveness of this gene-editing system for the treatment of PKU.

Hepatitis B virus (HBV) is a serious threat to people’s health, and long-term treatment with drugs such as interferon may lead to a significant increase in viral resistance.^263^ Moreover, these treatments do not eliminate HBV covalently closed circular DNA (cccDNA), and targeted destruction of cccDNA using Cas9 is an effective method for treating HBV. Wang et al. designed an infrared light-responsive bionanoparticle for delivery of the CRISPR/Cas9 system to HBV-infected cells.^264^ This device effectively inactivated HBV cccDNA. CRISPR-based editing technology has shown significant efficacy in treating a variety of liver diseases and is an effective strategy for future treatment of these diseases.

#### Cardiovascular diseases

Cardiovascular disease is one of the major causes of death in humans.^265,266^ Common cardiovascular diseases include atherosclerosis, myocardial hypertrophy, heart attack, and aortic dissection.^267–271^ However, unlike tumors and liver diseases, blood flow in the heart and blood vessels is faster and blood pressure is greater, posing a challenge for nanoparticle enrichment at the lesion site^272^ (Fig. 6c).

During fetal development, the number of cardiomyocytes expands rapidly, whereas cardiomyocytes gradually lose their ability to proliferate with aging.^273^ Although evidence of cardiomyocyte renewal has been obtained in many mammals, restoring the loss of cardiomyocytes caused by cardiomyopathy is not sufficient. Restoring the expression of genes associated with cardiomyocyte proliferation, such as myocyte enhancer factor 2 D (Mef2d) and Krüppel-like factor 15 (Klf15), in cardiomyocytes of adult animals may exert a positive effect on curing cardiomyocyte-related diseases.^274,275^ Direct delivery of CRISPRa systems into cardiomyocytes is relatively difficult and may also lead to widespread off-target effects. Schoger et al. first constructed a dCas9/VPR transgenic mouse, and this sequence was inserted after the myosin heavy chain (Myh) 6 promotor and transcribed in concert with Myh6.^276^ Since Myh6 is a cardiomyocyte-specific gene, expression of the dCas9/VPR system occurs only in cardiomyocytes.^277^ The subsequent injection of AAV9 carrying sgRNA activates the transcription of cardiomyocyte proliferation-related genes in the cells. Using this approach, a cardiomyocyte-specific expression activation system was obtained, and the timing of transcriptional activation was controlled, providing an important reference for cardiovascular studies or studies of other organs or tissues that are difficult to access directly.

The development of aortic disease is usually accompanied by the inflammation of vascular endothelial cells and the phenotypic transformation of smooth muscle cells in the vascular mesoderm.^278,279^ Zhang et al. constructed a hydroxyl-rich lipid nanoparticle capable of delivering CRISPR/Cas9 to vascular smooth muscle cells.^280^ In another study, Zhao et al. combined lipid nanoparticles and polymer nanoparticles to construct a delivery vehicle for endothelial cells.^238^ Although the CRISPR-based gene drug was successfully delivered to VSMCs and endothelial cells, the nanoparticles were still taken up by the liver in large quantities.

In addition, CRISPR/Cas9 technology is also widely used for bone regeneration and the treatment of CFTR, Alzheimer’s disease, obesity, and other diseases^72,73,192,281–285^ (Fig. 6d).

### FDA-approved clinical treatments and diagnostics

#### SARS-CoV-2 detection

Coronaviruses can cause life-threatening respiratory infections in humans and have caused three epidemics in the 21st century: severe acute respiratory syndrome coronavirus (SARS-CoV), Middle East respiratory syndrome coronavirus (MERS-CoV), and still-unbeaten SARS-CoV-2.^286–288^ All three highly pathogenic viruses belong to Betacoronavirus; however, high pathogenicity is not the greatest difficulty for humans to overcome. The susceptibility of humans to SARS-CoV-2, its ease of transmission, and its long incubation period make this virus difficult to eradicate. Therefore, the development of a test that rapidly detects SARS-CoV-2 in patients is more important than a treatment. RT‒PCR technology is a PCR method developed specifically for RNA detection that is convenient and reliable and is the current gold standard for detection. However, this assay requires a rigorous, high-level testing platform, which limits the number of people who can be tested.^289,290^ Antigen antibody assays have also been used for SARS-CoV-2 detection but are costly and not suitable for large-scale application. Therefore, a new low-cost method that does not require an instrumental platform must be developed.

SHERLOCK is an RNA detection tool previously constructed by Zhang Feng et al. based on Cas13a.^182^ However, SHERLOCK detection relies on multiple steps of RNA extraction and liquid handling, which may easily lead to cross-contamination or even infection of the assay personnel if not performed properly. The improved STOP (SHERLOCK testing in one pot) method based on SHERLOCK simplifies the detection method and increases the sensitivity of virus detection using isothermal amplification (LAMP).^184,291^ LAMP operates at 55–70 °C, and thus the heat-tolerant Cas12b from *Alicyclobacillus acidiphilus* (AapCas12b) became the protein of choice for STOP.^292^ Researchers then adopted the magnetic bead purification method to obtain RNA and concentrated the collected samples into the STOPCovid reaction mixture, which further shortened the detection time by directly using magnetic beads to adsorb RNA. Finally, STOPCovid.v2 was developed as the detection solution for SARS-CoV-2 based on CRISPR technology. In clinical assays, the method achieved a sensitivity of 93.1% and specificity of 98.5%, which were higher than the values of RT‒PCR. STOPCovid.v2 is a remarkable breakthrough that requires only a few simple instruments to perform the assay, and the results are easily distinguishable using test strips. It may become a “sharp sword” for humans to overcome SARS-CoV-2.

#### Sickle cell disease and β-thalassemia

Sickle cell disease (SCD) and transfusion-dependent β-thalassemia (TDT) are both caused by mutations in the hemoglobin β subunit gene and are among the most common single-gene genetic disorders worldwide.^293,294^ Sickle cell disease is characterized by an imbalance in the hemoglobin chain and hemolytic anemia, which is usually treated by blood transfusion and iron-chelation therapy. Patients with β-thalassemia have sickle-shaped red blood cells that carry less oxygen and usually experience pain, and thus they are usually treated in the clinic with hydroxyurea, pain relievers, and blood transfusions. Bone marrow transplantation has also been used to treat both diseases, but matching is difficult.^74^ When the pathogenesis of these two hematological diseases was studied at the genetic level, the transcription factor BCL11A was identified as a suppressor of fetal hemoglobin and γ-bead protein expression, and maintaining high levels of expression of these two proteins alleviated the symptoms of sickle cell disease and β-thalassemia.^295^

In 2019, Wu et al. used CRISPR/Cas9 to cleave the BCL11A enhancer sequence in HSCs and successfully downregulated its expression without inducing significant side effects.^296^ In December 2020, clinical data were released for a gene therapy called CTX001, a one-time therapy for SCD and TDT developed by CRISPR Therapeutics in association with Vertex Pharmaceuticals.^74^ This clinical trial used Cas9 to cleave the BCL11A enhancer region in hematopoietic stem and progenitor cells (HSPCs), causing them to lose enhancer activity. This technique reduced the expression of BCL11A and restored the production of γ-hemoglobin and fetal hemoglobin. Subsequently, the researchers transplanted the edited HSPCs into two patients who had SCD and TDT. The follow-up study found that fetal hemoglobin levels exhibited a substantial elevation in both patients at 12 months postinjection. At the final follow-up visits after 18 and 15 months, both patients had achieved normal fetal hemoglobin levels. Subsequent treatment of eight patients yielded similar results to the first two patients, indicating the general applicability and efficiency of this strategy. However, this method is not absolutely harmless, as both of the initial patients experienced varying degrees of adverse effects, which were not life-threatening and resolved after treatment. In another clinical study for the treatment of SCD, an approach using RNAi to knock down BCL11A was used.^297^ They constructed a lentiviral vector carrying short hairpin RNA (shRNA) and used this lentivirus to transduce CD34 + cells from SCD patients, and clinical success was also achieved.

#### Transthyretin amyloidosis

Transthyretin (TTR) amyloidosis is an autosomal dominant disorder mainly caused by the deposition of amyloid fibrils around cells that mainly threatens the human nervous system and heart.^298–300^ In the normal state, TTR monomers are synthesized in the liver to form tetrameric complexes that are involved in the transport of thyroid hormones. Mutant TTRs do not stabilize the tetrameric structure and dissociate before reassembling into amyloid fibrils. However, patients with TTR amyloidosis do not show significant symptoms of thyroid hormone deficiency, suggesting that TTR may not be a major carrier of thyroid hormones and that reducing TTR expression may be a possible approach to treat this disease.^298,301^ The main clinical treatment options are liver transplantation and stabilization of tetramers with the small molecule tafamidis; however, the latter is not a stable and effective approach.^31^ The FDA has also approved the siRNA drug patisiran for the treatment of this disease. Patisiran blocks the translation of TTR to slow the disease process, but repeated injections are required throughout the patient’s life.^302,303^

Since low TTR expression has no significant side effects, adopting a modality that permanently eliminates the mutated TTR gene may be able to eradicate TTR amyloidosis. In June 2021, Gillmore et al. reported the results of clinical trials for the in vivo delivery of a CRISPR-based gene-editing drug named NTLA-2001.^31^ NTLA-2001 consists of a liver-targeting LNP encapsulating sgRNA against the TTR gene and mRNA for SpCas9. This LNP has been used several times to carry gene drugs for delivery to the liver.^304,305^ In preliminary experiments in animal models, NTLA-2001 showed efficient permanent knockdown. Six patients were selected for treatment in this trial, and all patients received the drug injection without adverse effects during the treatment course. On the seventh day of receiving the drug, the patients’ blood indicators and liver function indicators were within normal limits. Three patients received a dose of 0.1 mg per kg, and the other three received a dose of 0.3 mg per kg to determine the efficacy of NTLA-2001. On day 28, 47%, 52%, and 56% reductions in blood TTR concentrations were detected in the three patients who received the low dose and 80%, 84%, and 96% reductions were detected in the three patients who received the high dose. This finding indicated that the efficacy of NTLA-2001 is dose-dependent and highly successful. A few months later, the method was granted orphan drug designation by the FDA, a recognition not only of NTLA-2001 but also of the in vivo delivery of CRISPR-based gene therapy.

#### Others

In 2019, Maeder et al. developed a genome-edited therapy (EDIT-101) to treat Leber congenital amaurosis type 10 (LCA10).^306^ They used an AAV5 vector loaded with saCas9 and sgRNA targeting the CEP290 mutant intron to deliver this gene-editing system into photoreceptor cells via a subretinal injection to delete or inactivate the mutated intron and restore normal expression of CEP290. The first clinical dosing of EDIT-101 was completed in March 2020. In September of the same year, clinical results for EDIT-101 showed that of the two groups receiving different doses, the mid-dose group experienced a more pronounced therapeutic effect, but the low-dose group experienced a poor therapeutic effect. Fortunately, none of the patients showed any serious adverse effects.

Human immunodeficiency virus (HIV) is a retrovirus that integrates into the host genome after infection and follows replication.^307^ Antiretroviral therapy (ART) has shown good results in curbing HIV replication and improving immune function, but ART only controls the progression of HIV.^308,309^ The HIV genome must be removed from the human genome to completely cure HIV infection. In 2020, Mancuso et al. reported the results of a study using AAV to deliver CRISPR/Cas9 in nonhuman primates for the treatment of HIV, and the results revealed that it is a viable strategy.^310^ In September 2021, the FDA approved a CRISPR gene-editing technology-based therapy for the treatment of HIV infection (EBT-101).

In addition, in August 2022, the FDA approved a clinical application for CRISPR therapy CRD-TMH-001 for the treatment of Duchenne muscular dystrophy (DMD).

**Fig. 7:**
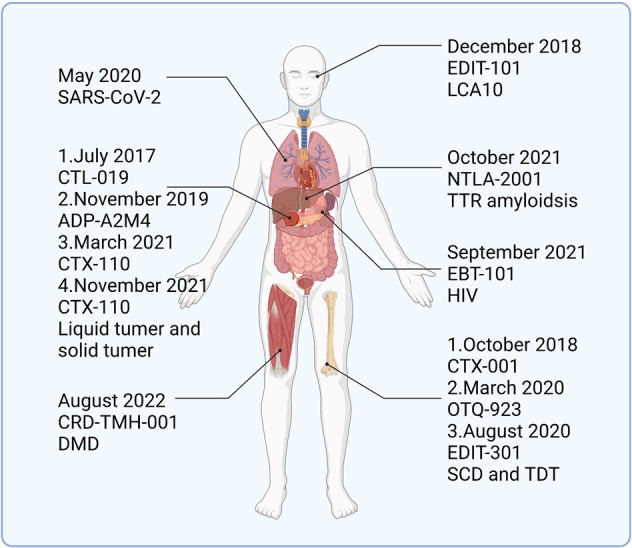
Summary chart of FDA-approved CRISPR therapies that can be used in clinical treatments. The text includes the date of FDA approval, the name of the therapy, and the type of applied diseases. DMD Duchenne muscular dystrophy, SCD sickle cell disease, TDT transfusion-dependent β-thalassemia, LCA10 Leber congenital amaurosis type 10, TTR transthyretin. (Figure was created with Biorender.com)

Together, these results suggest that the construction of an efficient, safe and stable targeting vector to deliver the CRISPR‒Cas9 system to the body is a promising new approach to treating diseases. Therefore, a number of delivery vehicles have been designed and manufactured for targeted delivery to disease sites according to the specific characteristics of a disease. These drugs show a good loading capacity and good prospects for clinical translation. Future research directions include improving vector targeting and designing more efficient CRISPR gene-editing systems according to the type of disease (Fig. 7).

## Limitations and challenges

The targeted delivery of CRISPR/Cas gene drugs to the body has the potential to treat diseases both in the laboratory and in the clinic. The advantages of a high specificity, effectiveness, and ease of handling make it one of the most sought-after technologies of the future. However, researchers have discovered some unexpected conditions when using CRISPR technology to edit genes.

### Limitations of CRISPR/Cas9

#### Off-target effects

Base mismatches between sgRNA and nontarget sequences may lead to off-target effects. The introduction of one or even multiple unknown mutations while repairing one error is clearly unacceptable.^311–313^ When sgRNA binds to the DNA strand, the seed sequence at the proximal end of the PAM binds to the target strand strictly according to base complementary pairing. The distal three to five bases sometimes do not detach as imagined when mismatching occurs but form an unusual duplex conformation under a strong force.^314,315^ This mechanism that allows mismatches may have arisen from the evolution of bacteria to counteract mutations in invading phages. Methods such as whole-genome sequencing and GUIDE-Seq have been developed to detect the occurrence of off-target effects.^316,317^ The persistent expression of Cas9 in large numbers of cells increases the likelihood of off-target effects, and controlling Cas9 activation may reduce their occurrence (Table 2).

Improving the specificity of sgRNAs and detaching them from the DNA strand when mismatches occur is the key to solving this problem. The REC3 domain of Cas9 is critical for sensing mismatches arising at the distal end of the PAM, and researchers have mutated the REC3 domain to detect which variants might improve the accuracy of Cas9.^318^ High-fidelity mutants such as HypaCas9 and Cas9-HF1 were rationally designed, and these mutants substantially improved the accuracy of Cas9. However, the interaction of mutated REC3 with the PAM-distal duplex is weakened, reducing the efficiency of Cas9. Bravo et al. found that the mismatch at bases 18–20 would form Ruv loop structure, which stabilized mismatch formation. They mutated all the residues involved in stabilization and obtained a Cas9 variant (SuperFi-Cas9) with a 500-fold reduction in the efficiency of DNA duplex cleavage at the 18th–20th base mismatch of sgRNA without affecting sgRNA-mediated double-strand cleavage with fully complementary bases. The addition a segment of a specialized structure to sgRNA to increase its specificity is also feasible. Kocak et al. designed a segment of hairpin structure at the 5′ end of sgRNA; this structure reduces the energy during mismatch and prevents the formation of an R-loop when mismatch occurs.^319,320^ The R-loop is necessary for Cas9 activation, and thus this structure also prevents DNA duplex cleavage in the presence of mismatches. In conclusion, off-target effects are the greatest obstacle to the widespread application of CRISPR-based gene-editing technology, and modifying the sgRNA with Cas9 to make it more specific might prevent the occurrence of unknown mutations.

#### Validity

When CRISPR/dCas9 carrying activating or repressing structural domains is used to regulate gene expression, the upregulation or knockdown of the gene might not be sufficient to achieve a therapeutic effect. The CRISPRa system is divided into two parts: the sgRNA/Cas9 complex, which plays a targeting role, and the activating structural domain, which enhances transcription.^59,321^ In general, when performing gene editing, only one sgRNA targeting the target site will be designed. Maeder et al. designed sgRNAs at four positions near the transcriptional start site of the target gene to obtain higher gene activation efficiency.^322^ The transcriptional activation efficiency of multiple sgRNAs exerted a certain synergistic effect, and higher efficiency was obtained when more sgRNAs were present. Moreover, the transcriptional activation efficiency of sgRNAs at each position is not the same but is strongly linked to the cell and gene.

The initial transcriptional activation domain is the VP64 or p65 activation structural domain formed by the complex of four transcribed VP16, and the activation of this structure is not strong. Tanenbaum et al. constructed a synthetic system composed of a structure that contains a polypeptide chain that can recruit up to 24 copies of the protein to obtain higher activation efficiency.^148^ The structure was used to recruit multiple copies of VP64 to form the dCas9-SunTag-VP64 transcriptional activation system. In a study of the activation of cell cycle suppressor cyclin-dependent kinase inhibitor 1B (CDKN1B), dCas9/VP64 did not affect cell cycle progression, whereas the same sgRNA carrying dCas9-SunTag-VP64 significantly inhibited cell cycle progression and reduced cell growth. VP64, p65 and Rta have been reported to have the ability to activate transcriptional. Chavez et al. used dCas9/VP64 as a backbone and added p65 and Rta to construct the transcriptional complex dCas9-VP64-p65-Rta (dCas9/VPR) as a transcriptional activation system.^145^ With its simple structure and high efficiency, dCas9/VPR is one of the most frequently used activation systems for targeted delivery of CRISPRa. Other transcriptional activation systems, such as dCas9/SAM, dCas9/SPH and dCas9/VP192, are also able to substantially increase the efficiency of gene activation.^323–325^ The activation or repression of a gene is related to many conditions, such as the location of the sgRNA, the selection of the effector structural domain, the type of cell and the targeted gene (Table 4).

**Table 4:** Efficiency of CRISPRa- and CRISPRi-mediated activation and inhibition of different genes

CRISPR activation interference	Carrier system	Target genes	Target cells	Regulation efficiency in vitro	Regulation efficiency in vivo	Ref.
dCas9/VPR	SPDS	Cnr1	Primary cortical neurons	3-fold		^140^
	SPDS	DANCR	rASC	400,000-fold		^192^
	SPDS	DANCR	rBMSC	600-fold	5-fold	^192^
	DPDS	GDNF	U2OS	2.5-fold	15-fold	^209^
	DPDS	Cnga1	661 W	6.3-fold		^188^
	DPDS	Opn1mw	MEF	1.9-fold		^188^
	SPDS	CFTR	Human nasal cells	3–4-fold	5-fold	^72^
	DPDS	Mef2d	Cardiomyocytes	30-fold	3-fold	^276^
	DPDS	Klf15	Cardiomyocytes	15-fold	4-fold	^276^
	SPDS	HGF	HEK293T	10–15-fold	1.8-fold	^220^
dCas9/VP64	DPDS	HNF4α	AML12	40-fold	1.2-fold	^261^
	DPDS	CT45	A2780	4-fold		^349^
	SPDS	miR-524	LN-229	4-fold	3-fold	^206^
	SPDS	miR-524	MDA-MB-23	7-fold	3-fold	^206^
dCas9/VP160	SPDS	Lama1	C2C12 myoblasts	14,000-fold		^350^
dCas9/KRAB	SPDS	Fabp4	Adipocytes	0.4-fold	0.3-fold	^285^
	SPDS	PCSK9	AML12	0.3-fold	0.5-fold	^186^
dCas9/Epigenetic regulators	DPDS	DUX4	FSHD myocytes	0.3–0.6-fold	0.6–0.8-fold	^139^

*SPDS* single plasmid delivery system, *DPDS* dual plasmid delivery system

#### Applicability

CRISPR/Cas9 can theoretically target any position in the genome but is limited to PAM sequences, which prevents Cas9 from reaching certain positions.^326,327^ In particular, when using the base-editing tools CBEs or ABEs, the edited bases are located at specific relative positions of the PAM sites, and CBEs or ABEs may not be able to perform the base change function without a suitable PAM site.^26^ Researchers have worked to modify Cas9 to ensure that it is not restricted to PAMs, and they have obtained multiple variants of Cas9 that are not restricted to recognizing NNG by mutating the Cas9 site or adding modified structural domains (Table 1).

Walton et al. mutated multiple amino acid sites of Cas9 to obtain a SpCas9 variant (SpRY) that is almost free from PAM restriction.^117^ They first constructed a purine-rich PAM site, and SpRY was able to achieve partial gene editing at the site where the PAM is NRN (R is A or G) without lower editing efficiency than wild-type SpCas9. Next, they constructed pyrimidine-rich PAM loci, before which almost all Cas9 variants failed to recognize C- or T-rich loci, and SpRY exerted a gene-editing effect on 13 of the 31 loci constructed. Although SpRY still exhibits a stronger bias toward G-rich PAM sites, targeting pyrimidine-rich PAM sites using SpRY carrying a base-editing effector also promotes efficient base substitution. SpRY without the restriction of PAM is more prone to off-target effects. The previously reported Cas9-HF variant is effective at avoiding off-target effects, and after mutating the same site, SpRY-HF1 is able to eliminate almost all off-target effects.^108^

Modifying Nme2Cas9, a Cas9 variant from *Neisseria meningitidis*, to recognize a wider variety of PAM sequences is a promising approach.^328,329^ Compared to SpCas9, Nme2Cas9 is smaller and has greater potential for targeted delivery, and Nme2Cas9 also has strong gene-editing activity in mammalian cells. Researchers established a new selection platform to screen for Nme2Cas9 variants that recognize a single specified base. The final screen yielded four reliable variants: eNme2-C and eNme2-C.NR, eNme2-T.1 and eNme2-T.2. Compared with SpRY, these variants are not only PAM-independent but are also smaller in size. Except for eNme2-C.NR, the other three mutations exhibited stronger or similar gene-editing efficiency and fewer off-target effects. In conclusion, freeing Cas9 from the restriction of PAM sites may satisfy the need for single-base editing, the targeted cleavage of double strands, and other methods, which are important for the treatment of diseases caused by single-gene mutations.

#### Chromosomal disorganization

The safety of CRISPR-based gene-editing technology is a key topic of concern for researchers. The cleavage of double-stranded DNA by Cas9 usually triggers NHEJ repair, and these repaired DNA strands are usually missing a few base pairs or have a few added base pairs, which is the expected result. However, when verifying editing efficiency, researchers found that massive base deletions and chromosomal structural translocations sometimes occurred.^320,330–332^ These errors may lead to positional diseases such as malignant tumors and are obviously not acceptable in clinical applications, although the probability of their occurrence is low.^110,333^

The repeated cleavage of target genes by CRISPR/Cas9 is one of the important causes of chromosomal translocations and deletions. Yin et al. combined an exonuclease structural domain with Cas9 to reduce the occurrence of these mutations.^110^ This structure performs end processing immediately upon the completion of cleavage, reducing the likelihood of producing intact ends. This approach effectively prevents perfect repair of the DNA strand and thus duplicate cleavage of the genome by Cas9. The authors fused spCas9 with optimized three-prime repair exonuclease 2 (TREX2) to generate a Cas9 exonuclease (Cas9TX). In experiments with engineered T cells and other cells, Cas9TX was clearly able to suppress chromosomal translocations relative to the high-fidelity SpCas9 variant.

### Limitations of targeted delivery

#### Deviation from the desired position

Viral and nonviral vectors are usually delivered to animals by systemic administration, and the vectors make CRISPR gene drugs immune to blood and tissue degradation. However, unmodified vectors are susceptible to capture by metabolic organs in the body. The CRISPR/Cas system does not lose activity when entering nontarget cells but genetically modifies healthy cells, which may lead to unpredictable consequences. Improved delivery vehicles are necessary to reduce the entry of gene drugs into nontarget cells. Approaches to improve delivery functionality include covering the carriers with a biofilm or adding peptides recognized by target cell receptors.^232,334,335^ Designing environmentally responsive nanoparticles according to the target organ microenvironment enhances gene drug enrichment, such as variations in pH, reactive oxygen species (ROS), and adenosine triphosphate (ATP) levels.^33^ The nanomaterial shell disintegrates in a specific environment, exposing the core, which then enters the cell through endocytosis. However, when the microenvironment in certain diseased tissues does not differ significantly from that of other tissues, constructing a nanoparticle that is induced by multiple conditions to release its contents is a feasible method for disease-specific targeting. In addition, light-, magnetic-, and ultrasound-responsive CRISPR/Cas9 delivery systems have been developed to support precision delivery.^33^ When applied to the treatment of human diseases, the administration of drugs by in situ injection prevents them from being transported in the blood flow throughout the body. Regulating the expression of target genes may require a more modest CRISPRa or CRISPRi approach, and the changes imposed by CRISPR/dCas9-based transcriptional regulatory systems are reversible compared to altering genomic sequences to silence genes.^59,143^

#### Biocompatibility

Suitable vectors must be constructed for candidate cells to reduce the possibility of adverse reactions caused by off-target CRISPR/Cas9 systems. The complex process from entry to function requires that the vector must be biocompatible, have a high encapsulation ability, and be able to traverse the cell membrane.^336^ The immune response resulting from delivery of the material into the body must also be considered when designing the system. Commonly used Cas9 proteins derived from *S. pyogenes* and *S. aureus* have been reported to trigger an immune response in humans. As a method to overcome this challenge, a modified Cas9 lacking response-causing exons was delivered via AAV to effectively avoid humoral and cellular immune responses in juvenile and adult mice.^30^ Moreover, even the modified Cas9 must also be transported in a vector designed to avoid triggering the host immune response. In vivo, viruses, lipids, and exosomes are effective at avoiding immune clearance, whereas synthetic chemical nanoparticles require a protective coating on the surface, such as modified PEG, which also stabilizes the polymer in the blood environment, or the inclusion of modified CD47 protein.^33,337,338^ Furthermore, plant exosomes are more likely to escape detection by the immune system due to their natural origin. The use of plant exosomes for delivering CRISPR/dCas9 systems is also more acceptable for safety reasons due to the large differences between plant and mammalian pathogens. However, research on the delivery of gene drugs by plant exosomes is not yet mature, especially as many plants produce exosomes with different characteristics.^339,340^ Overall, the development of additional delivery vehicles with low immunogenicity along with surface-modified proteins or polypeptides that effectively prevent the vehicle from being cleared by the immune system is necessary to facilitate the targeted delivery of gene drugs in the clinic, and the natural resistance of plant exosomes to immune clearance and their low pathogenicity highlighting their bright application prospects for this purpose.

## Outlook and conclusions

After decades of development, CRISPR/Cas is no longer limited to cleaving DNA strands but has spawned a large family of single-base gene editing, transcriptional regulatory, and RNA strand cutting approaches. Thus, these systems may be applied to treat most human diseases, such as cancer, chronic diseases, and genetic diseases caused by a single gene. In cells, CRISPR/Cas9 and other systems have shown unparalleled gene-editing capabilities. However, the development of a safe, stable, and efficient strategy for delivering gene-editing tools to diseased cells in vivo is a major challenge to their use in clinical applications. CRISPR is usually delivered as plasmids, mRNA, or RNP, but all three forms are immunologically cleared both from the blood and from the digestive system.

AAV is one of the most commonly used vectors for delivering gene drugs, and even without considering loading capacity, the integration of AAV into the human genome may lead to disease and may be difficult to accept. Nonviral vectors with higher loading capacities and good safety profiles have also become ideal for delivery, especially LNPs, which have been used in clinical trials. Since vectors such as LNPs and AuNPs are not derived from organisms, they often trigger immunogenicity in vivo. These vectors are also susceptible to erroneous uptake by the digestive organs, although researchers have added modifications or peptides to prevent immune clearance. Exosomes from organisms or biofilms obtained as nonviral vector masks are very effective in avoiding immune clearance, and the proteins and peptides enriched in the biofilm may help the gene drug reach the target cells.

When a disease occurs, the microenvironment surrounding cells at the site of the disease is altered, which distinguishes it from normal tissue and creates favorable conditions for the development of delivery vectors. Disease onset is also accompanied by the overexpression of inflammatory factors in cells or other disease-associated membrane proteins. The addition of peptides that specifically recognize these membrane proteins allows nanomaterials to specifically recognize diseased cells and deliver CRISPR/Cas to the cells for gene-editing functions. In addition, the use of nanomaterials that respond to disease-specific conditions might also increase the enrichment of nanomaterials at the target site. Therefore, an in-depth study of the mechanism of disease onset, as well as the various pathways involved, the changes in the expression of various proteins, and the microenvironment in which the disease cells are located, may be very helpful in the construction of vectors for the targeted delivery of gene drugs.

In addition, the efficiency and safety of CRISPR/Cas9 itself are key facets to consider in clinical applications. Off-target CRISPR/Cas9 effects may lead to serious consequences, and these possible scenarios should be identified and improved before clinical application. Scientists have fully investigated the cleavage mechanism of Cas9 and developed several variants of Cas9 with a decreased likelihood of off-target effects and no reduction in efficiency. Off-target effects may be effectively avoided by modifying the sgRNA. In conclusion, in-depth studies of the mechanisms of disease occurrence, the development of more efficient and specific delivery vectors, and improvements in Cas9 variants with broader and safer adaptations are important. With these foundations, CRISPR/Cas technology will enter full clinical application to help treat human diseases.
